# Comparative Functional Effects of Abemaciclib and Arcyriaflavin A in *MYCN*-Amplified and Non-Amplified Neuroblastoma Cells: An Exploratory Transcriptomic Study

**DOI:** 10.3390/ijms27167349

**Published:** 2026-08-17

**Authors:** Burcu Çerçi, Hatice İlayhan Karahan, Mustafa Soyöz, Melek Pehlivan, İbrahim Pirim

**Affiliations:** 1 Department of Medical Biology, Faculty of Medicine, Izmir Katip Celebi University, Cigli, Izmir 35620, Türkiye; haticeilayhan.karahan@ikcu.edu.tr (H.İ.K.); mustafa.soyoz@ikcu.edu.tr (M.S.); ibrahim.pirim@ikcu.edu.tr (İ.P.); 2 Vocational School of Health Services, Izmir Katip Celebi University, Cigli, Izmir 35620, Türkiye; melek.pehlivan@ikcu.edu.tr

**Keywords:** neuroblastoma, *MYCN*, CDK inhibition, Abemaciclib, Arcyriaflavin A, exploratory transcriptomics

## Abstract

Cyclin-dependent kinase (CDK) inhibitors are promising therapeutic agents for neuroblastoma, particularly in *MYCN*-amplified tumors; however, the molecular responses associated with CDK inhibition remain incompletely understood. *MYCN*-amplified BE(2)-C and *MYCN*-non-amplified SH-SY5Y neuroblastoma cells were treated with Abemaciclib or Arcyriaflavin A. Drug responses were evaluated using MTT, Annexin V ELISA, wound healing, colony formation, quantitative real-time PCR, and Western blot analyses. Exploratory transcriptomic profiling was performed by RNA sequencing. Because only one biological replicate was available per condition, transcriptomic analyses were limited to descriptive expression trends and pathway-oriented interpretation rather than statistical differential expression analysis. Both inhibitors reduced cell viability in a dose- and time-dependent manner, with BE(2)-C cells showing greater sensitivity than SH-SY5Y cells. Treatment also increased Annexin V levels, reduced wound closure, impaired clonogenic growth, and decreased CDK4/CDK6 expression, as confirmed by qRT-PCR and Western blotting. Exploratory transcriptomic profiling identified concordant expression trends in genes associated with cell-cycle regulation, DNA replication, and mitotic progression, with broader pathway modulation observed in *MYCN*-amplified cells. Overall, CDK inhibition suppresses proliferation-associated phenotypes in neuroblastoma and is accompanied by exploratory transcriptomic changes that identify candidate pathways for future validation and mechanistic investigation of *MYCN*-associated therapeutic responses.

## 1. Introduction

Neuroblastoma is the most common extracranial solid malignancy of childhood and accounts for a disproportionate number of pediatric cancer-related deaths despite multimodal treatment strategies [1]. Patients with high-risk disease continue to experience poor clinical outcomes owing to rapid tumor progression, metastatic dissemination, and the development of therapeutic resistance [2]. Among the molecular features associated with aggressive neuroblastoma, amplification of the *MYCN* oncogene is one of the strongest prognostic indicators and is linked to enhanced proliferation, metabolic reprogramming, genomic instability, and unfavorable survival [3].

Aberrant activation of cyclin-dependent kinase (CDK) signaling is a critical driver of uncontrolled proliferation in *MYCN*-amplified neuroblastoma [4]. The CDK4/6–RB–E2F regulatory axis controls G1/S cell-cycle progression by promoting RB phosphorylation and subsequent activation of E2F-dependent transcription [5]. In *MYCN*-driven tumors, this pathway is further reinforced through functional cooperation between *MYCN* and E2F transcription factors, creating an increased dependence on intact cell-cycle regulatory mechanisms [6,7]. Consequently, pharmacological inhibition of CDKs has emerged as an attractive therapeutic strategy for targeting proliferative vulnerabilities in neuroblastoma.

Abemaciclib and Arcyriaflavin A were selected to provide an exploratory comparison between two compounds that differ in their clinical development and kinase selectivity profiles. Abemaciclib is a clinically approved, comparatively well-characterized CDK4/6 inhibitor that has also demonstrated preclinical activity in neuroblastoma models [8,9]. Arcyriaflavin A is a natural, preclinical compound with a broader kinase-inhibition profile and reported antiproliferative, pro-apoptotic, and migration-inhibitory effects in cancer cells [10,11]. Their comparative evaluation was therefore intended to investigate whether compounds with distinct kinase-targeting profiles produce different functional and molecular responses in *MYCN*-defined neuroblastoma models.

Beyond their direct effects on proliferation, accumulating evidence indicates that CDK inhibitors influence multiple cellular processes, including apoptosis, differentiation, DNA damage responses, and transcriptional regulation [12,13]. Previous studies have demonstrated the sensitivity of neuroblastoma cells to CDK inhibition by examining individual inhibitors and selected cell-cycle or viability-related endpoints [4,13]. However, direct comparisons of CDK-targeting compounds with different kinase selectivity profiles in *MYCN*-amplified and *MYCN*-non-amplified neuroblastoma models remain limited. Moreover, the relationship between inhibitor-induced functional responses and broader pathway-level transcriptional patterns has not been sufficiently characterized. Exploratory transcriptomic profiling may therefore help identify candidate biological pathways and gene-expression patterns associated with drug responses and generate hypotheses for subsequent mechanistic investigation [14]. The primary objective of the present study was to determine whether *MYCN* amplification status influences the functional response of neuroblastoma cells to CDK inhibition and to compare the effects of two CDK inhibitors with different kinase selectivity profiles, Abemaciclib and Arcyriaflavin A. To address this question, *MYCN*-amplified BE(2)-C and *MYCN*-non-amplified SH-SY5Y cells were evaluated using complementary assays of cell viability, apoptosis-associated responses, migration-related wound closure, clonogenic growth, and CDK4/CDK6 expression. Exploratory transcriptomic profiling was additionally performed to identify candidate pathway-level expression patterns associated with treatment response. Rather than establishing statistically validated differential expression, the transcriptomic analysis was intended to generate mechanistic hypotheses and guide future validation studies in *MYCN*-defined neuroblastoma.

## 2. Results

### 2.1. Cytotoxic Effects of CDK Inhibitors

**Figure 1:**
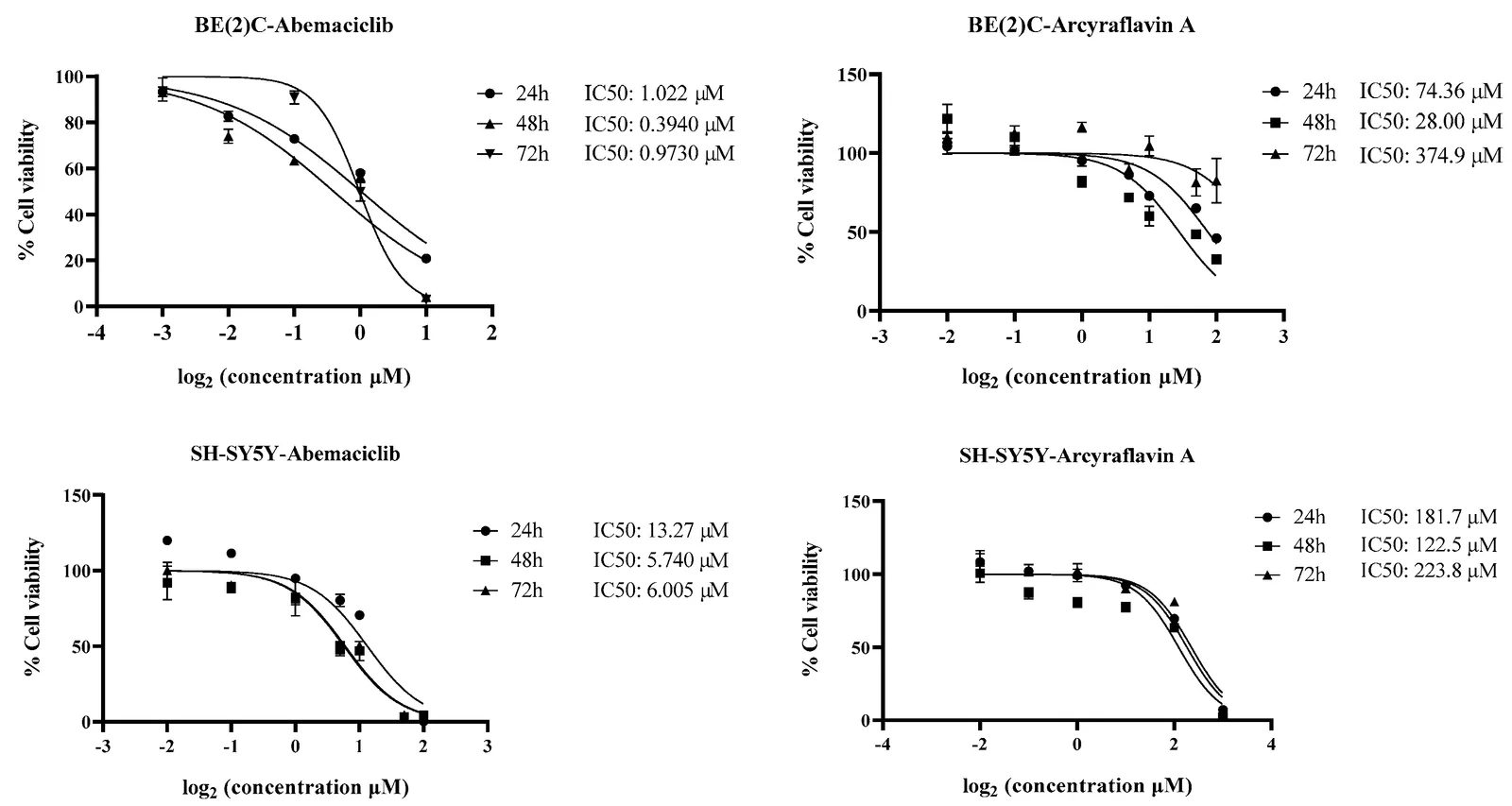
Cytotoxicity results at 24, 48 and 72 h after treatment with Abemaciclib and Arcyriaflavin A.

Cell viability assays revealed that both Abemaciclib and Arcyriaflavin A induced a dose- and time-dependent reduction in cell viability in SH-SY5Y and BE(2)-C neuroblastoma cell lines (Figure 1). Increasing concentrations of each compound resulted in a progressive decrease in metabolic activity, indicating effective cytotoxic responses. Notably, *MYCN*-amplified BE(2)-C cells exhibited significantly greater sensitivity to CDK inhibitors compared to *MYCN*-non-amplified SH-SY5Y cells. This enhanced susceptibility was evident across multiple concentrations and exposure times, suggesting a heightened dependency of *MYCN*-positive cells on CDK-mediated cell cycle regulation for survival. Abemaciclib demonstrated a more pronounced cytotoxic effect relative to Arcyriaflavin A, particularly in BE(2)-C cells, consistent with its selective inhibition of CDK4/6 and established efficacy in *MYCN*-driven neuroblastoma models. In contrast, Arcyriaflavin A induced a more gradual but sustained reduction in cell viability, indicating potential differences in target engagement or downstream signaling effects.

### 2.2. CDK Inhibition Induces Apoptosis-Associated Cellular Responses

**Figure 2:**
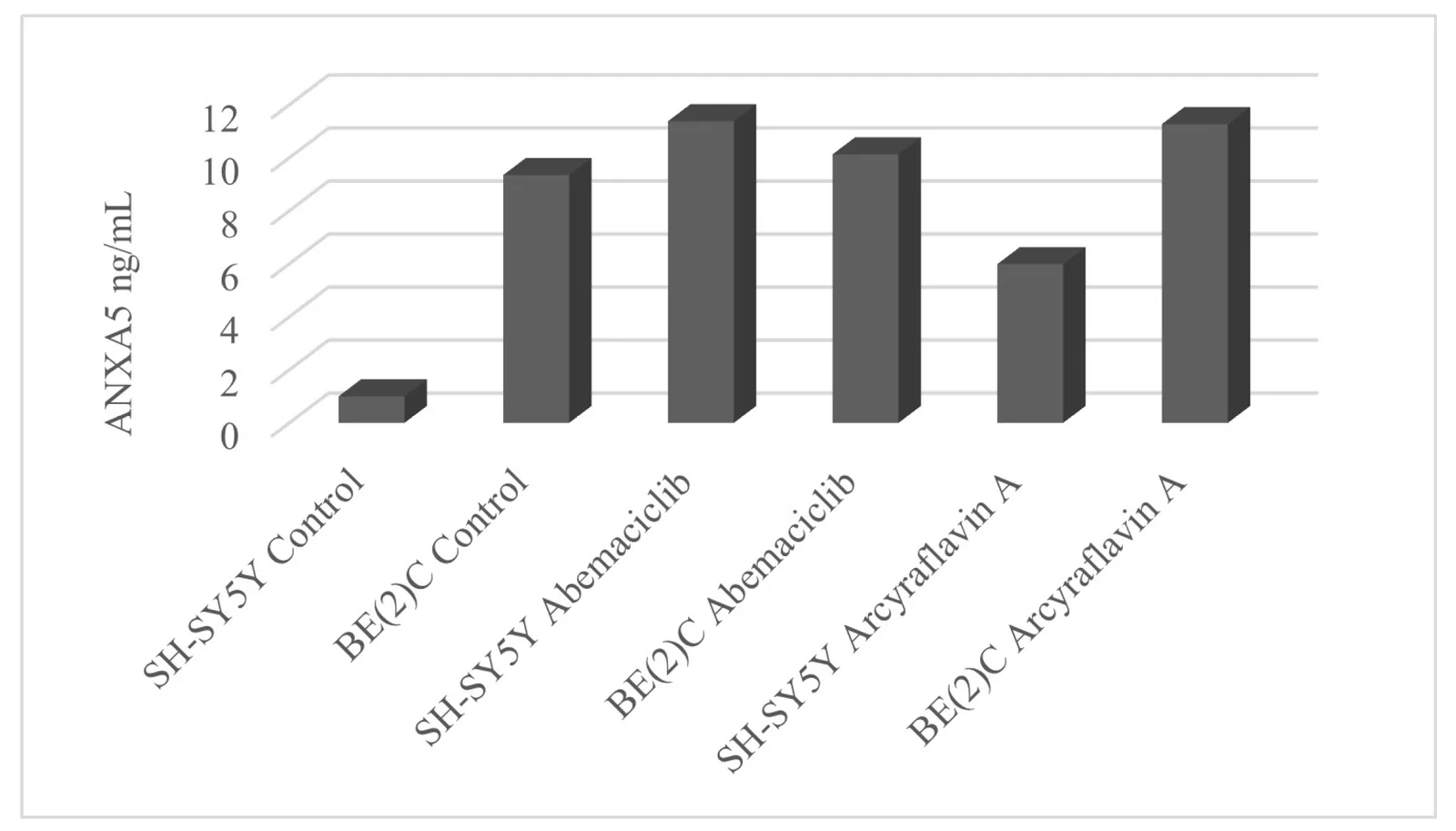
Annexin V (ANXA5) levels in neuroblastoma cells following CDK inhibitor treatment.

Annexin V (ANXA5) levels were quantified by ELISA to evaluate apoptosis-associated membrane alterations following Abemaciclib and Arcyriaflavin A treatment in *MYCN*-non-amplified SH-SY5Y and *MYCN*-amplified BE(2)-C neuroblastoma cells (Figure 2). In SH-SY5Y cells, basal ANXA5 levels were low in the control group, whereas treatment with Abemaciclib resulted in a marked increase in ANXA5 concentration. Arcyriaflavin A treatment also elevated ANXA5 levels compared to control, although to a lesser extent than Abemaciclib. In BE(2)-C cells, control samples exhibited higher basal ANXA5 levels relative to SH-SY5Y controls. Both Abemaciclib and Arcyriaflavin A further increased ANXA5 levels in BE(2)-C cells, with Arcyriaflavin A producing the highest ANXA5 concentration among all experimental groups. Overall, treatment-induced increases in ANXA5 levels were more pronounced in *MYCN*-amplified BE(2)-C cells than in SH-SY5Y cells, indicating a stronger apoptosis-associated response to CDK inhibition in the *MYCN*-amplified neuroblastoma model.

### 2.3. CDK Inhibition Reduces Wound Closure and Clonogenic Growth

**Figure 3:**
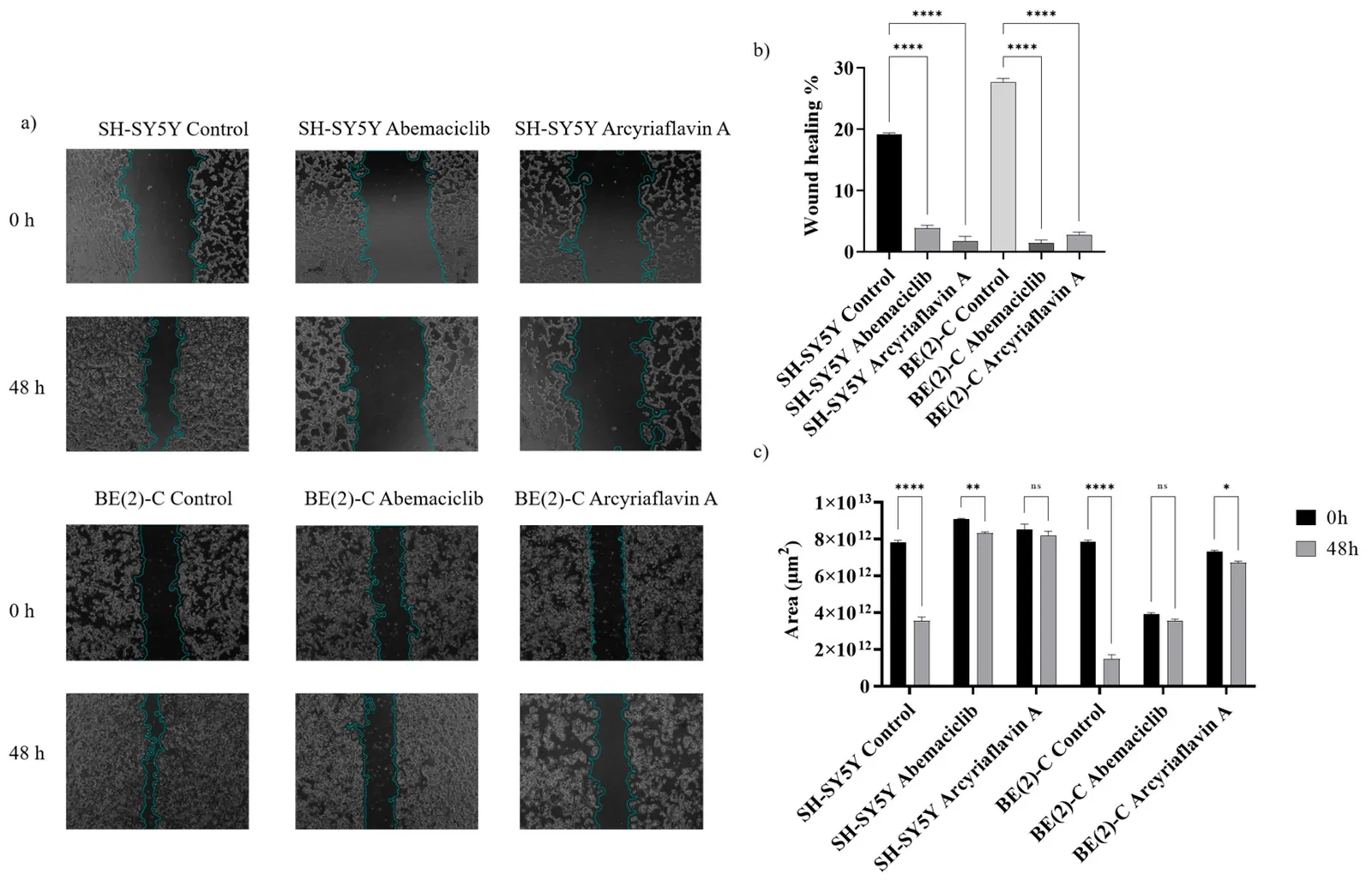
Effects of CDK inhibition on neuroblastoma cell migration assessed by wound healing assay. (**a**) Representative images show wound closure in SH-SY5Y and BE(2)C neuroblastoma cells under control conditions and following treatment with Abemaciclib or Arcyriaflavin A. (**b**,**c**) Bar graphs represent relative wound closure measurements derived from image-based analysis, illustrating reduced migratory capacity following CDK inhibitor treatment. ns, not significant; * *p* < 0.05; ** *p* < 0.01, *** *p* < 0.001, and **** *p* < 0.0001.

Cell migratory behavior following CDK inhibitor treatment was evaluated using a wound healing assay in SH-SY5Y and BE(2)-C neuroblastoma cell lines (Figure 3). In control groups, both cell lines exhibited substantial wound closure over the experimental period, indicating preserved migratory capacity. Treatment with Abemaciclib resulted in a visible reduction in wound closure compared to controls, suggesting impaired cell migration. A more pronounced inhibition of wound closure was observed following Arcyriaflavin A treatment, with wider residual wound areas remaining at the end of the assay. This inhibitory effect on migratory behavior was evident in both cell lines and appeared more marked in *MYCN*-amplified BE(2)-C cells. Quantitative analysis of wound closure supported qualitative observations, demonstrating reduced migration-associated wound closure following CDK inhibition, particularly in Arcyriaflavin A-treated groups.

**Figure 4:**
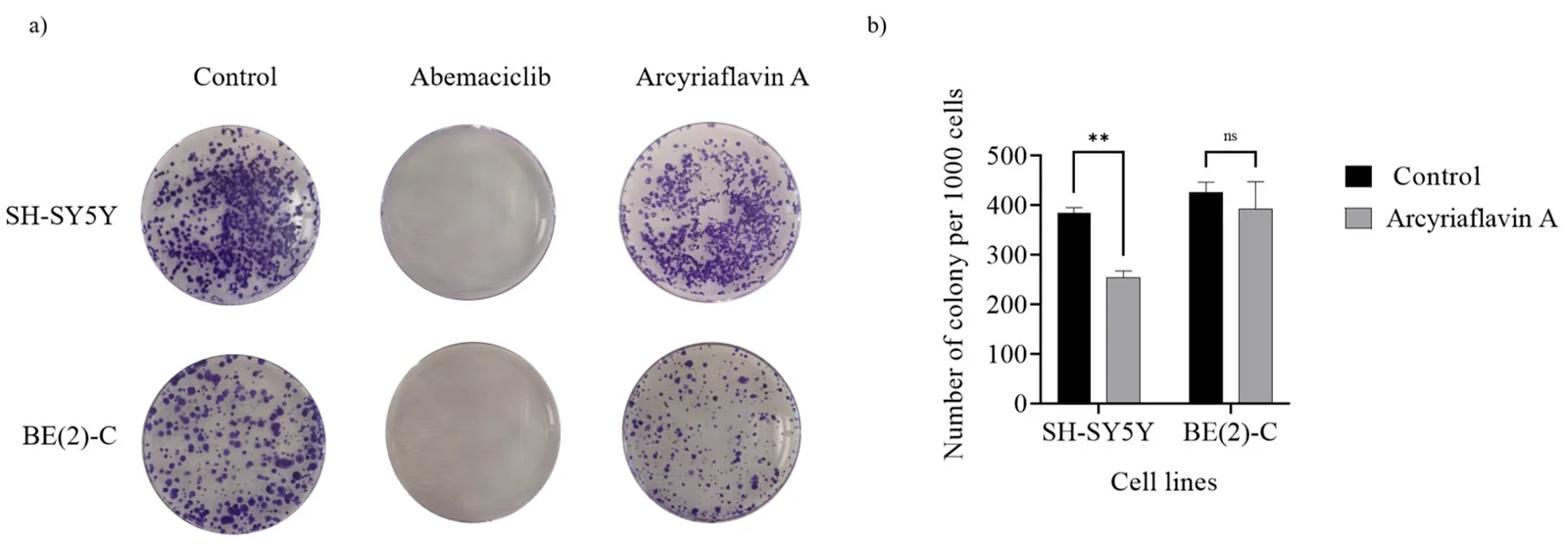
Effects of CDK inhibition on clonogenic growth of neuroblastoma cells. (**a**) Representative images of colony formation assays performed in SH-SY5Y and BE(2)-C neuroblastoma cell lines under control conditions and following treatment with Abemaciclib or Arcyriaflavin A are shown. (**b**) Bar graphs depict relative colony formation levels obtained from image-based quantification, illustrating reduced clonogenic growth following CDK inhibitor exposure. ns, not significant; ** *p* < 0.001.

The long-term proliferative capacity of neuroblastoma cells following CDK inhibitor treatment was assessed using a colony formation assay. Control groups in both SH-SY5Y and BE(2)-C cell lines formed dense and well-defined colonies, reflecting sustained clonogenic potential (Figure 4). In contrast, treatment with Abemaciclib resulted in a visible reduction in the number and size of colonies. Arcyriaflavin A treatment led to a more pronounced suppression of colony formation, with markedly fewer and smaller colonies observed. This reduction in clonogenic growth was evident across both cell lines and appeared more substantial in *MYCN*-amplified BE(2)-C cells. Quantitative colony measurements supported the observed decrease in clonogenic capacity following CDK inhibition, indicating impaired long-term proliferative potential in treated neuroblastoma cells.

### 2.4. Validation of CDK4/CDK6 Modulation

*CDK4* and *CDK6* transcripts in both SH-SY5Y and BE2-C neuroblastoma cell lines, with more pronounced effects observed in *MYCN*-amplified BE2-C cells. Quantitative real-time PCR analysis confirmed the reduction in *CDK4* and *CDK6* mRNA expression following CDK inhibition across both cell lines, with Abemaciclib inducing stronger suppression compared to Arcyriaflavin A (Table 1).

**Figure 5:**
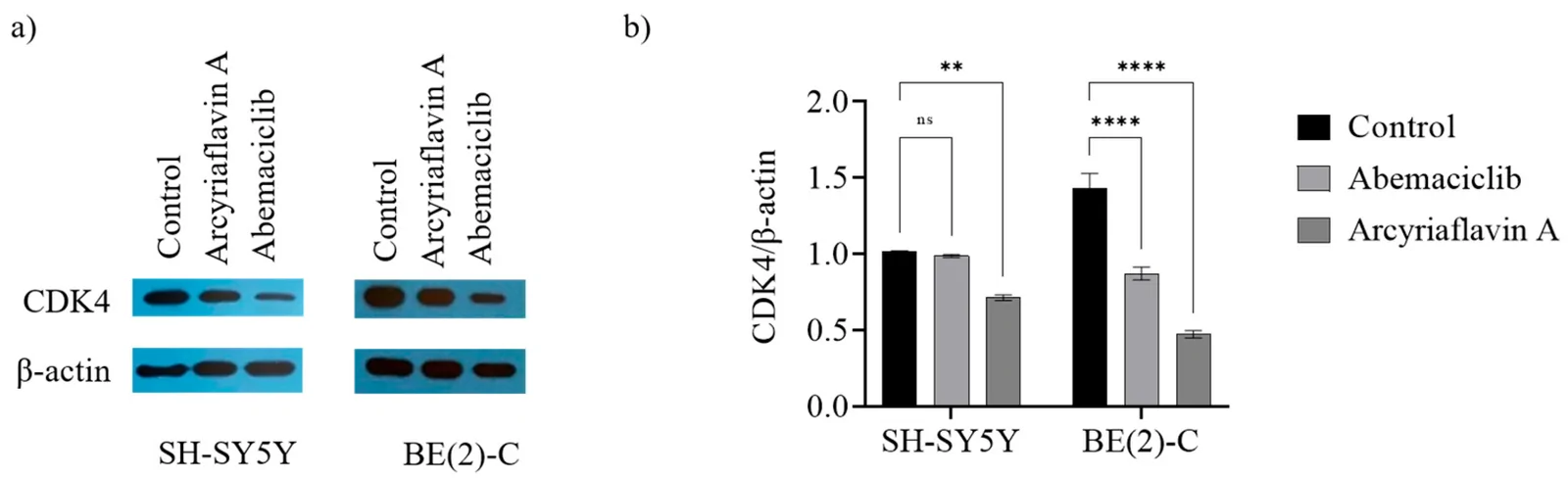
Validation of CDK4 protein expression following CDK inhibitor treatment in *MYCN*-defined neuroblastoma cells. (**a**) Representative Western blot images showing CDK4 protein expression in SH-SY5Y and BE(2)-C neuroblastoma cells under control conditions and following treatment with Abemaciclib or Arcyriaflavin A. β-actin was used as the loading control. (**b**) Densitometric analysis of CDK4 protein expression normalized to β-actin. Relative protein expression levels were quantified using ImageJ software (version 1.54p) and are presented as mean ± SD. Statistical significance was determined by one-way ANOVA followed by the appropriate multiple-comparison test. ** *p* < 0.01; **** *p* < 0.0001; ns, not significant.

To assess whether transcriptional changes were reflected at the protein level, Western blot analysis was performed for CDK4 (Figure 5). In control groups, baseline expressions of the analyzed proteins were detectable in both cell lines. Treatment with Abemaciclib resulted in a visible reduction in target protein levels compared to controls, indicating modulation of CDK-associated signaling pathways. Arcyriaflavin A treatment produced a more pronounced decrease in protein expression, particularly in *MYCN*-amplified BE(2)-C cells. Densitometric analysis supported these observations, revealing lower relative protein abundance following CDK inhibitor exposure. Overall, the Western blot findings corroborate the functional assay results, suggesting that CDK inhibition alters signaling pathways associated with proliferation and survival in neuroblastoma cells, with a stronger effect observed in the *MYCN*-amplified model.

### 2.5. Exploratory Transcriptomic Profiling Identifies Candidate Biological Pathways Associated with CDK Inhibition

To complement the functional characterization of CDK inhibition, exploratory transcriptomic profiling was performed in SH-SY5Y and BE(2)-C neuroblastoma cells following treatment with Abemaciclib or Arcyriaflavin A. After quality filtering and annotation-based transcript curation, 29,690 protein-coding transcripts were retained for downstream exploratory analyses. Gene expression changes were summarized descriptively using log_2_ fold-change values calculated relative to untreated controls. Because RNA sequencing was performed using a single biological replicate for each experimental condition, statistical differential expression analysis was not conducted, and all transcriptomic observations should therefore be interpreted as exploratory. Across all treatment groups, expression changes were observed in genes associated with multiple biological processes. Rather than focusing on genome-wide transcript ranking, subsequent analyses concentrated on predefined biological pathways relevant to CDK signaling, cell-cycle regulation, DNA replication, and MYCN biology. This targeted strategy enabled biological interpretation of expression trends while avoiding overinterpretation of individual transcript changes.

Overall, exploratory transcriptomic profiling suggested that CDK inhibition was accompanied by widespread modulation of genes involved in proliferative signaling. Although these observations cannot establish statistically validated differential expression, they provide candidate molecular pathways that may contribute to the functional responses observed in neuroblastoma cells following CDK inhibitor treatment.

### 2.6. Exploratory Expression Trends in Cell-Cycle Regulatory Pathways

**Figure 6:**
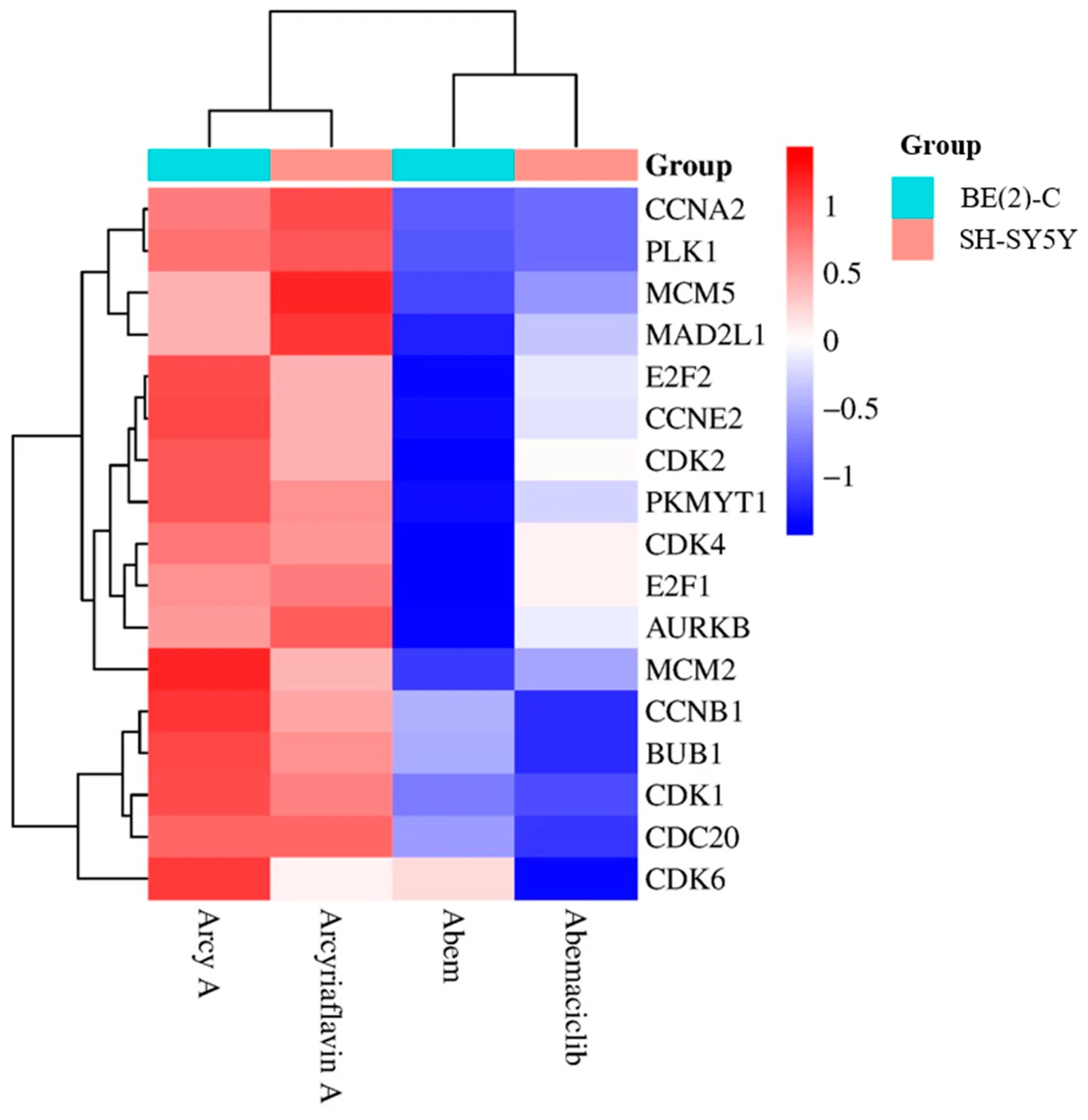
Exploratory expression trends of selected cell cycle-associated genes following CDK inhibitor treatment. Heatmap illustrating relative log_2_ fold-change (log_2_FC) values of selected genes involved in the CDK–RB–E2F signaling axis, DNA replication, and mitotic checkpoint regulation in *MYCN*-amplified BE(2)-C and *MYCN*-non-amplified SH-SY5Y cells following IC_50_ treatment with Abemaciclib or Arcyriaflavin A. Color intensity represents relative expression trends compared with untreated controls, with red indicating higher and blue indicating lower expression. Because RNA sequencing was performed using a single biological replicate per condition, the heatmap provides an exploratory visualization of candidate expression patterns and should not be interpreted as statistically validated differential gene expression.

To explore molecular processes potentially associated with the observed antiproliferative effects, expression trends were examined within predefined cell-cycle-related gene sets. Across both neuroblastoma cell lines, genes involved in CDK signaling, G1/S transition, DNA replication licensing, and mitotic progression generally showed reduced expression following treatment with Abemaciclib and Arcyriaflavin A. Similar directional changes were observed for several cyclins, E2F transcription factors, DNA replication regulators, and mitotic checkpoint components (Figure 6 and Table S1).

Among these candidate genes, expression trends involving *CDK1*, *CDK2*, *CDK4*, *CDK6*, *CCNA2*, *CCNB1*, *CCNE2*, *E2F1*, *E2F2*, *CDC6*, members of the MCM family, *PLK1*, *AURKB*, and *CDC20* were consistent with reduced activity of proliferative pathways following CDK inhibition. These observations were generally more pronounced in *MYCN*-amplified BE(2)-C cells than in SH-SY5Y cells. Because these analyses were based on exploratory transcriptomic profiling without biological replication, the observed expression trends should not be interpreted as statistically validated differential expression. Instead, they identify candidate biological processes that are consistent with the functional effects observed in cell viability, apoptosis-associated responses, and clonogenic assays, and provide a framework for future mechanistic validation.

### 2.7. Exploratory Network and Pathway Analyses

**Figure 7:**
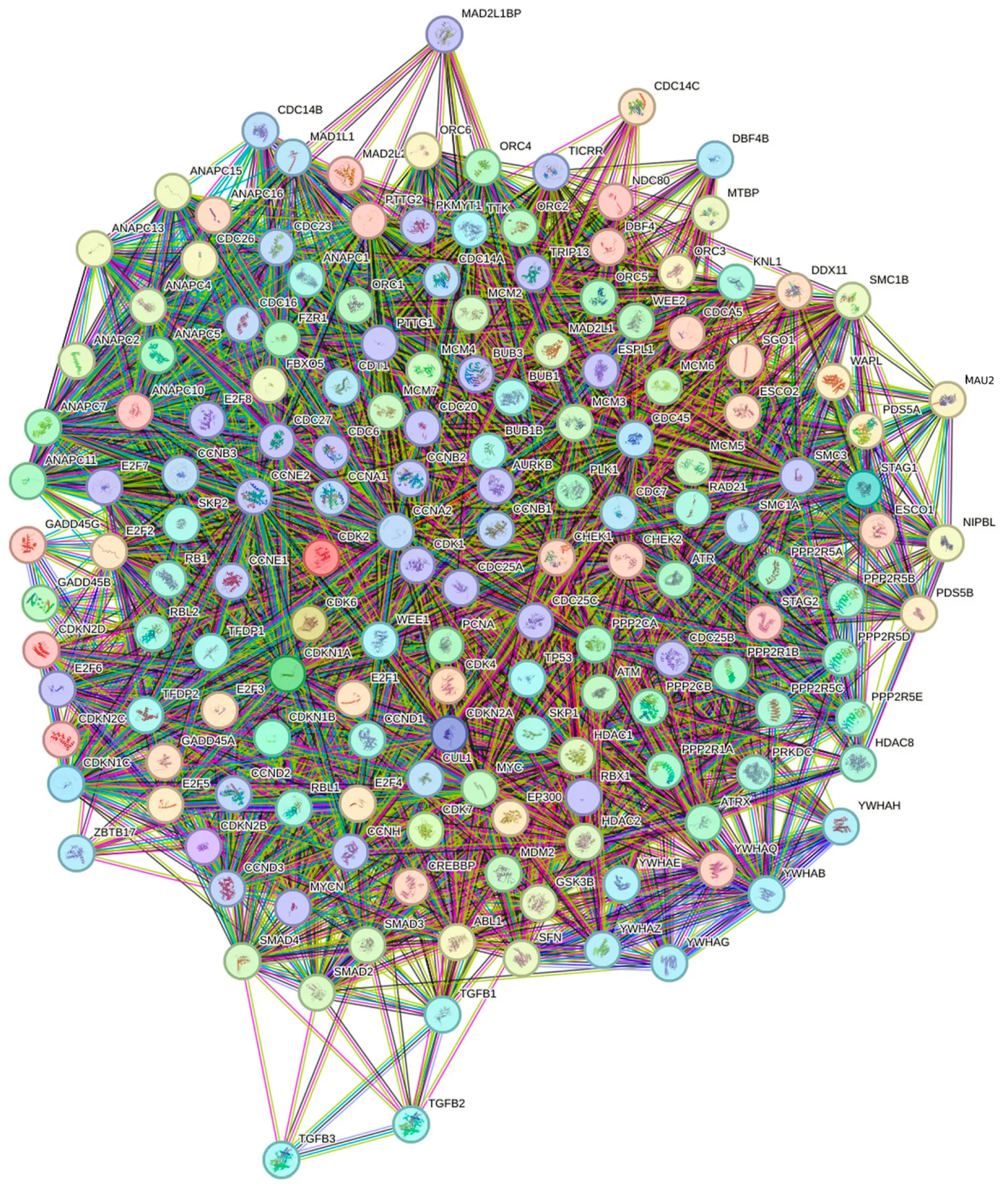
STRING protein–protein interaction network of cell cycle-associated genes.

**Figure 8:**
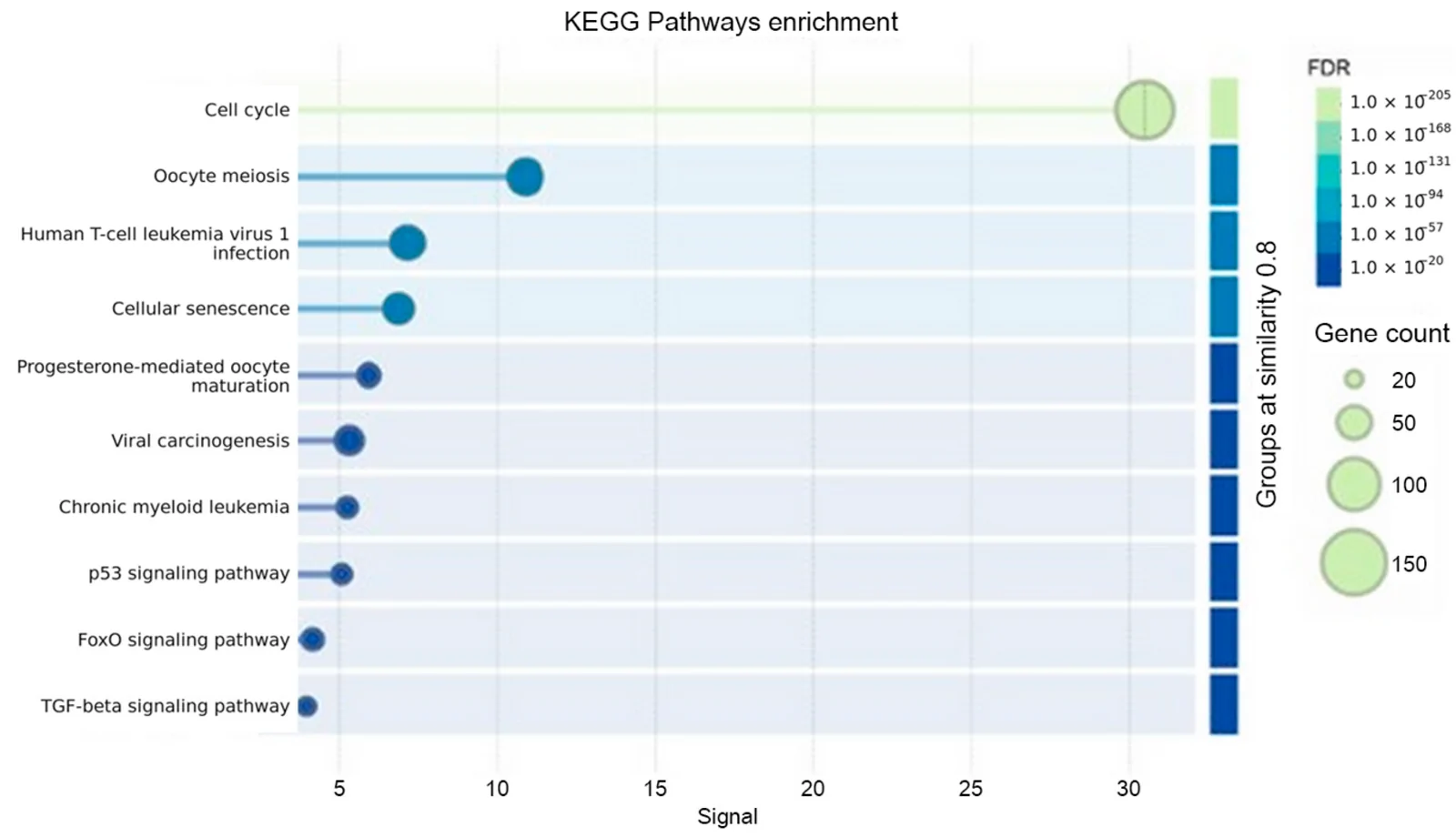
Exploratory KEGG pathway-enrichment analysis of candidate genes showing descriptive expression changes following CDK inhibitor treatment.

To further examine the biological relationships among the candidate genes identified in the exploratory transcriptomic dataset, protein–protein interaction and pathway enrichment analyses were performed. STRING analysis indicated that many of the selected genes were represented within established cell-cycle regulatory networks, including CDKs, cyclins, E2F transcription factors, DNA replication proteins, and mitotic checkpoint regulators (Figure 7 and Table S1). KEGG, Gene Ontology, and Reactome analyses similarly highlighted biological categories related to cell-cycle regulation, DNA replication, and mitotic progression (Figure 8 and Supplementary Figures S2–S10).

These network analyses were intended to facilitate biological interpretation of the exploratory transcriptomic observations rather than to provide independent statistical validation. Collectively, they suggest that genes showing concordant expression trends after CDK inhibition participate in biologically related regulatory pathways that may contribute to the phenotypic responses observed in neuroblastoma cells.

Bubble plot showing exploratory KEGG pathway-enrichment results derived from candidate cell cycle-associated genes showing descriptive expression changes following CDK inhibitor treatment. Dot size represents gene count, and color intensity represents the enrichment metric generated by the analysis. The highlighted pathways include cell cycle, DNA replication, p53 signaling, and cellular senescence. Because the candidate genes were derived from RNA-sequencing data obtained using one biological replicate per condition, these enrichment results should be interpreted as hypothesis-generating and not as evidence of statistically validated differential expression or mechanistic pathway regulation.

## 3. Discussion

Cyclin-dependent kinases (CDKs) are central regulators of cell-cycle progression, and their dysregulation contributes to uncontrolled proliferation in many malignancies, including neuroblastoma [15]. Pharmacological inhibition of CDK activity has therefore emerged as an attractive therapeutic strategy, particularly in *MYCN*-driven tumors where the RB–E2F signaling axis is highly active [16,17]. In the present study, we evaluated the biological effects of two mechanistically distinct CDK inhibitors, Abemaciclib and Arcyriaflavin A, in *MYCN*-amplified BE(2)-C and *MYCN*-non-amplified SH-SY5Y neuroblastoma cells. By integrating functional assays with exploratory transcriptomic profiling, we sought to characterize the cellular responses associated with CDK inhibition and identify candidate molecular pathways that may contribute to these responses.

Both Abemaciclib and Arcyriaflavin A reduced cell viability in a dose- and time-dependent manner, with BE(2)-C cells exhibiting greater sensitivity than SH-SY5Y cells. These findings are consistent with previous studies demonstrating that *MYCN*-amplified neuroblastoma cells display increased dependence on CDK4/6-mediated cell-cycle progression and are therefore more susceptible to CDK inhibition [17]. *MYCN* cooperates with E2F transcription factors to sustain proliferative signaling, and disruption of this regulatory network has been shown to preferentially impair the growth of *MYCN*-driven tumors [4,7]. Our findings further support the concept that MYCN status influences cellular responsiveness to CDK-targeted therapies.

Although both inhibitors reduced cell viability, distinct response patterns were observed. Abemaciclib produced a more pronounced reduction in metabolic activity, whereas Arcyriaflavin A exerted stronger effects in wound closure and colony formation assays. Similar differences have been reported between selective CDK4/6 inhibitors and compounds with broader CDK inhibitory profiles, suggesting that inhibition of different kinase subsets may result in distinct biological consequences despite producing comparable antiproliferative activity [18]. These observations indicate that evaluation of CDK inhibitors should extend beyond short-term viability assays to include complementary functional analyses reflecting different aspects of tumor cell behavior.

The phenotypic and molecular effects observed in this study should not be attributed exclusively to CDK4/6 inhibition. Although Abemaciclib is primarily regarded as a CDK4/6-directed inhibitor, kinome-profiling studies have demonstrated activity against additional CDKs, particularly *CDK9*, as well as non-CDKs, including members of the *PIM*, *HIPK*, *DYRK*, *CaMKII*, and *GSK-3* families [19,20]. Arcyriaflavin A also exhibits a broader kinase-related activity profile, with reported inhibitory activity against *cyclin D1/CDK4*, *cyclin B/CDK1*, *cyclin E/CDK2*, and *CaMKII* [21,22]. In addition, its anticancer effects have been associated with modulation of GSK-3β signaling [10]. Consequently, engagement of these additional targets and pathways may have contributed to the distinct effects of the two compounds on metabolic activity, apoptosis-associated responses, wound closure, clonogenic growth, and transcriptional patterns. The present findings should therefore be interpreted as compound-level responses associated with CDK-directed kinase inhibition rather than as evidence of effects mediated exclusively through *CDK4* and *CDK6*.

Annexin V analysis demonstrated increased apoptosis-associated membrane alterations following treatment with both inhibitors. The response was particularly evident in *MYCN*-amplified BE(2)-C cells, supporting previous reports that CDK inhibition promotes apoptotic signaling in high-risk neuroblastoma [23,24]. Interestingly, Arcyriaflavin A induced comparatively strong Annexin V responses despite showing less pronounced effects on metabolic activity than Abemaciclib. Similar observations have been described previously and may reflect differences in the kinetics and mechanisms underlying CDK inhibitor-induced cellular responses [25].

The wound healing and colony formation assays further demonstrated that both CDK inhibitors influenced aggressive cellular characteristics of neuroblastoma cells. Treatment with Abemaciclib and Arcyriaflavin A resulted in reduced wound closure compared with untreated controls, with Arcyriaflavin A producing the more pronounced effect in both *MYCN*-amplified and *MYCN*-non-amplified cells. Previous studies have suggested that CDK inhibition affects cellular processes associated with tumor progression in addition to its established role in cell-cycle regulation [26,27,28,29]. Similarly, colony formation assays revealed a marked reduction in clonogenic survival following treatment with both inhibitors, indicating impaired long-term proliferative capacity. Notably, although Arcyriaflavin A exhibited a weaker effect on short-term metabolic activity than Abemaciclib, it produced a stronger reduction in wound closure and clonogenic growth. Together, these findings suggest that different CDK inhibitors may differentially influence multiple phenotypic characteristics of neuroblastoma cells while maintaining a common antiproliferative effect.

At the molecular level, the reduction in *CDK4* and *CDK6* expression observed by quantitative real-time PCR was supported by Western blot analysis, confirming that the transcriptional changes were accompanied by decreased CDK4 protein abundance. Although Abemaciclib is a selective CDK4/6 inhibitor, both compounds reduced *CDK4* expression, suggesting that inhibition of CDK signaling may also influence regulatory feedback mechanisms controlling CDK expression [30]. The consistency observed between RNA sequencing, qRT-PCR, and protein analysis strengthens the reliability of the validated molecular findings and supports the biological relevance of CDK pathway modulation in both neuroblastoma models.

To complement the validated functional and molecular findings, exploratory transcriptomic profiling was performed to investigate biological processes potentially associated with CDK inhibitor responses. These exploratory transcriptomic observations provide biological context for the validated phenotypic findings and generate candidate hypotheses for future investigation; however, they do not establish causal or mechanistic relationships between individual expression changes and the observed cellular responses.

Rather than serving as a statistically powered differential expression analysis, the transcriptomic dataset was intended to provide a pathway-oriented overview of expression trends that could support biological interpretation and generate hypotheses for future investigation. Within this framework, genes associated with cell-cycle regulation, DNA replication, mitotic progression, and RB–E2F signaling exhibited broadly concordant expression trends following treatment with both CDK inhibitors. These observations are consistent with the established role of CDK inhibition in restricting proliferative signaling and support the functional changes observed in cell viability, clonogenic growth, and apoptosis-associated assays.

Exploratory pathway interrogation further indicated that several components of the CDK–RB–E2F regulatory network, including cyclins, E2F transcription factors, DNA replication licensing proteins, and mitotic checkpoint regulators, displayed coordinated expression patterns following treatment. Previous studies have demonstrated that inhibition of CDK activity suppresses transcriptional programs governing cell-cycle progression and DNA replication [31,32]. Accordingly, the expression trends observed in the present study are biologically consistent with the known mechanisms of CDK inhibition. Nevertheless, because transcriptomic profiling was performed using a single biological replicate per condition, these findings should be interpreted as candidate molecular signatures requiring confirmation in future studies incorporating biological replication and statistical differential expression analysis.

Comparison of the two neuroblastoma models suggested that expression changes within proliferation-associated pathways were generally more pronounced in *MYCN*-amplified BE(2)-C cells than in *MYCN*-non-amplified SH-SY5Y cells. This observation parallels the greater functional sensitivity of BE(2)-C cells to both CDK inhibitors and is consistent with previous reports describing enhanced dependence of *MYCN*-driven neuroblastoma on CDK-mediated proliferative signaling [4,17]. *MYCN* amplification has been shown to reinforce E2F-dependent transcription and replication-associated gene expression, thereby increasing susceptibility to pharmacological disruption of the CDK–RB axis [7,32]. Although the present transcriptomic analysis does not permit statistical comparison of global gene expression, the concordance between the functional phenotype and exploratory expression patterns suggests that MYCN status may influence the biological response to CDK inhibition.

Network-based analyses using STRING and functional enrichment approaches provided additional biological context for the exploratory transcriptomic observations. Candidate genes exhibiting concordant expression trends were predominantly associated with interconnected pathways regulating cell-cycle progression, DNA replication, and mitotic control. These analyses were not intended to validate differential expression but rather to facilitate biological interpretation by placing individual gene expression changes within established regulatory networks. Such pathway-oriented analyses are increasingly used to generate mechanistic hypotheses that can subsequently be examined using targeted molecular approaches.

Several limitations should be considered when interpreting the present findings. Abemaciclib was used in this study as a pharmacological model of CDK4/6-directed inhibition and not as an established standard-of-care treatment for neuroblastoma. Its selection was based on the biological dependence of *MYCN*-driven neuroblastoma on CDK-associated cell-cycle signaling and previously reported preclinical activity in neuroblastoma models. Nevertheless, the absence of a standard-of-care comparator limits direct conclusions regarding the relative therapeutic efficacy of the tested compounds. Most importantly, transcriptomic profiling was performed using a single biological replicate for each experimental condition. Consequently, statistical differential expression analysis and false discovery rate estimation were not feasible, and the transcriptomic findings should therefore be regarded as exploratory. In contrast, the principal conclusions of the present study are supported by multiple complementary experimental approaches, including cell viability analysis, apoptosis-associated measurements, clonogenic assays, quantitative real-time PCR, and Western blotting. Together, these independent experimental observations provide consistent evidence that CDK inhibition suppresses proliferative characteristics of neuroblastoma cells while identifying candidate transcriptional pathways that warrant further investigation.

## 4. Materials and Methods

### 4.1. Cell Lines and Culture

Human neuroblastoma cell lines SH-SY5Y (ATCC^®^ CRL-2266™) and BE(2)-C (ATCC^®^ CRL-2268™) were used in this study. Both cell lines are archived in the American Type Culture Collection (ATCC) database under accession/catalog numbers CRL-2266 and CRL-2268, respectively. The authenticated cell stocks used in this study were obtained from the Cell Culture Collection of İzmir Katip Çelebi University (İKÇÜ), Department of Medical Biology. SH-SY5Y is a thrice-cloned subline of the SK-N-SH neuroblastoma cell line originally established from a metastatic bone tumor of a 4-year-old patient, whereas BE(2)-C is a MYCN-amplified human neuroblastoma cell line established from a patient with neuroblastoma.

The *MYCN*-amplified BE(2)-C cell line and the *MYCN*-non-amplified SH-SY5Y cell line were maintained in DMEM/F-12 supplemented with 10% FBS, 1% Penicilin-Streptomycin and 1% L-glutamine (all from Gibco, Thermo Fisher Scientific, Waltham, MA, USA). Both cell lines were passaged upon reaching approximately 80% confluence and were used for downstream cell culture experiments, as previously described [13].

### 4.2. Cytotoxicity Assay

BE(2)-C and SH-SY5Y cells were seeded into 96-well plates at a density of 5 × 10^3^ cells/100 µL and incubated for 24 h to allow cell attachment. Arcyriaflavin A and Abemaciclib were purchased from MedChemExpress (Monmouth Junction, NJ, USA). Subsequently, Arcyriaflavin A and Abemaciclib (0.1–100 µM) were added to the wells, and after 24, 48, and 72 h of incubation, 5 mg/mL of MTT (BioFroxx, Einhausen, Germany) solution was applied to each well. Following 4 h of incubation, the formazan crystals were dissolved using DMSO (Tekkim, Bursa, Turkiye), and absorbance was measured at 570 nm. To evaluate the cytotoxic effects of Arcyriaflavin A on healthy cells, HEK293 cells were also treated with 0.1–100 µM of Arcyriaflavin A, and the cytotoxic dose was determined at 24, 48, and 72 h using the MTT assay [13].

### 4.3. Wound Healing Analysis

The assay was performed under serum-free conditions to reduce the contribution of cell proliferation; however, because no proliferation-blocking agent was used, the results were interpreted as changes in wound closure rather than as an exclusive measure of cell migration. SH-SY5Y and BE(2)-C cells were seeded into 6-well plates at a density of 8 × 10^5^ cells per well and incubated for 24 h to allow cell attachment. Once the cells reached approximately 90% confluence, the culture medium was removed, and the cells were gently washed with serum-free DMEM. A straight scratch was created using a 200 µL pipette tip, and detached cells were removed by washing twice with serum-free DMEM. The cells were then treated with IC_50_ concentrations of Arcyriaflavin A and Abemaciclib. Images of the wounded areas were captured at 0, 24, and 48 h, and cell migration was evaluated by comparing the closure of the scratch area over time [33].

### 4.4. Colony Formation Analysis

The colony formation ability of neuroblastoma cells under the effects of Abemaciclib and Arcyriaflavin A was evaluated. Following crystal violet staining, colonies containing at least 50 cells were counted manually by visual inspection. Neuroblastoma cells were seeded into 6-well plates at a density of 1000 cells per well. After 24 h of incubation, cells were allowed to attach to the surface and were then treated with IC_50_ concentrations of Abemaciclib and Arcyriaflavin A for 24 h. Following treatment, the culture medium was removed, and the colonies were gently washed with PBS (Gibco, Thermo Fisher Scientific, Waltham, MA, USA) and fixed with cold methanol for 20 min. The cells were subsequently stained with 0.1% crystal violet, and the colonies were counted [34].

### 4.5. Determination of Annexin V Levels by ELISA

Annexin V quantification was performed using a commercial ELISA kit (Bioassay Technology Laboratory-BT LAB, Shanghai, China, Cat. No: E1107Hu) following the manufacturer’s instructions. Briefly, cells seeded at 1.5 × 10^4^ cells/well were treated with IC_50_ concentrations for 24 h. Post-treatment, cells were harvested and lysed via three freeze–thaw cycles in PBS, and the resulting supernatants were collected. Samples were incubated with anti-ANX-5 antibody and Streptavidin-HRP at 37 °C for one hour. After five washes, the reaction was developed with substrate solutions for 10 min in the dark, terminated with stop solution, and the optical density was measured at 450 nm [35].

### 4.6. RNA Isolation

Total RNA was isolated using the TRIzol reagent method. Briefly, cells seeded at 2 × 10^5^ cells/well were treated with IC_50_ concentrations of Abemaciclib and Arcyriaflavin A for 24 h. After lysis and chloroform-mediated phase separation, the aqueous phase was collected, and RNA was precipitated with isopropanol. The resulting pellet was washed with 75% ethanol, air-dried, and resuspended in RNase-free water [31]. RNA concentration and purity were quantified via NanoDrop spectrophotometry, and samples were normalized to 100 ng and stored at −80 °C for subsequent qRT-PCR and transcriptomic analyses.

### 4.7. Quantitative Real-Time PCR (qRT-PCR)

Complementary DNA (cDNA) synthesis was performed using RevertAid First Strand cDNA Synthesis Kit (Thermo Scientific, Waltham, MA, USA) according to the manufacturers’ instructions. Quantitative real-time PCR (qRT-PCR) analysis was carried out to determine the expression levels of the *CDK4* and *CDK6* genes using Ampliqon SYBR Green Master Mix (Odense, Denmark) in accordance with the manufacturer’s protocol. The housekeeping gene *GAPDH* was used as an internal reference for normalization. Primer sequences used for qRT-PCR analysis are listed in Table 2. Relative gene expression levels were calculated using the 2^−ΔΔCt^ method.

### 4.8. Western Blot

Western blot analysis was performed to evaluate protein expression levels. Briefly, cells seeded at 1.5 × 10^5^ cells/well were treated with IC_50_ concentrations of Arcyriaflavin A and Abemaciclib for 48 h. Following treatment, cells were lysed using RIPA buffer (Merck, Darmstadt, Germany), and protein concentrations were determined via Bradford assay. Equal amounts of protein (30 µg) were separated by SDS–PAGE and transferred onto PVDF membranes. After blocking with 5% non-fat dry milk, membranes were incubated overnight at 4 °C with primary antibodies against CDK4 (Elabscience Biotechnology Inc., Houston, TX, USA) and β-actin (Cell Signaling Technology, Danvers, MA, USA), followed by a 1.5-h incubation period with secondary antibodies. Protein bands were visualized using HRP (Merck, Darmstadt, Germany) substrate and radiographic film. Band intensities were quantified using ImageJ software (version 1.54p) and normalized to β-actin [36].

### 4.9. Exploratory Transcriptomic Profiling and Targeted Pathway Analysis

Total RNA extracted from SH-SY5Y and BE(2)-C neuroblastoma cells was evaluated for quality using NanoDrop spectrophotometry and an Agilent 2100 Bioanalyzer. Only RNA samples with an RNA Integrity Number (RIN) ≥ 8.0 were used for library preparation. RNA sequencing libraries were generated using the Illumina^®^ (San Diego, CA, USA) Stranded Total RNA Prep, Ligation with Ribo-Zero Plus kit (Illumina, San Diego, CA, USA, Cat. No. 20040529). Indexed libraries were sequenced on an Illumina (San Diego, CA, USA) platform using paired-end 150-bp reads, yielding approximately 30 million paired-end reads per sample. Raw sequencing data were processed by the sequencing service provider using standard quality-control procedures, including adapter trimming, removal of low-quality reads, alignment to the human reference genome (GRCh38), and transcript quantification. Gene expression values were reported as Transcripts Per Million (TPM), providing normalized estimates of transcript abundance.

Because RNA sequencing was performed using a single biological replicate for each experimental condition, statistical differential expression analysis could not be performed. Consequently, transcriptomic analyses were conducted within an exploratory and hypothesis-generating framework. Gene expression changes were evaluated descriptively by calculating log_2_ fold-change values between treated samples and their corresponding untreated controls. No statistical significance testing, false discovery rate correction, or differential expression inference was applied. To facilitate biological interpretation, transcripts corresponding to mitochondrial genes, ribosomal RNAs, transfer RNAs, long non-coding RNAs, small nucleolar RNAs, small nuclear RNAs, microRNAs, pseudogenes, and other non-protein-coding transcripts were excluded prior to downstream analyses. This filtering strategy was applied uniformly to all samples and was independent of expression magnitude.

Rather than performing unbiased genome-wide differential expression analysis, we adopted a hypothesis-driven pathway interrogation strategy focusing on biological processes known to be associated with CDK signaling and MYCN biology. Candidate genes were selected from established literature and curated pathway resources, including the Kyoto Encyclopedia of Genes and Genomes (KEGG) and Gene Ontology (GO), and organized into functional modules representing MYCN–RB–E2F signaling, cell-cycle regulation, G1/S transition, DNA replication licensing, mitotic progression, cell-cycle checkpoint regulation, and ubiquitin-mediated cell-cycle control. Expression trends within these predefined functional categories were examined descriptively to identify coordinated biological responses associated with CDK inhibition. Genes were interpreted according to both the direction and magnitude of log_2_ fold-change values. Genes exhibiting |log_2_FC| ≥ 1 were considered to show relatively pronounced expression changes and were highlighted for biological interpretation; however, this threshold was used solely for descriptive purposes and does not represent statistical differential expression.

To further explore potential functional relationships among candidate genes, protein–protein interaction (PPI) networks were generated using STRING (version 11.5) with a high-confidence interaction score (≥0.7). Functional enrichment analyses, including KEGG pathways and Gene Ontology categories, were performed to assist biological interpretation of the candidate gene sets. Because these analyses were based on exploratory transcriptomic observations obtained from single biological replicates, all enrichment results should be considered hypothesis-generating and require validation in independent biological experiments.

### 4.10. Statistical Analysis

All functional and molecular validation experiments, including MTT, Annexin V ELISA, wound healing, colony formation, qRT-PCR, and Western blot analyses, were performed in three independent biological experiments, with three technical replicates per experiment. RNA sequencing was performed using one biological replicate per experimental condition.

Statistical analyses were performed using GraphPad Prism version 9.4.1. Data obtained from functional assays were expressed as mean ± standard deviation (SD). Comparisons between experimental groups were performed using unpaired Student’s *t*-test or two-way ANOVA. A *p* value < 0.05 was considered statistically significant. Due to the presence of a single biological replicate in RNA sequencing experiments, transcriptomic analyses were conducted in a descriptive and exploratory manner without statistical differential expression testing. Gene expression changes were therefore interpreted based on log_2_ fold change (log_2_FC) values and pathway-level enrichment analyses rather than inferential statistical significance.

## 5. Conclusions

This study demonstrates that both Abemaciclib and Arcyriaflavin A exert significant antiproliferative effects in MYCN-defined neuroblastoma cells by reducing cell viability, increasing apoptosis-associated responses, suppressing wound closure, impairing clonogenic growth, and decreasing CDK4 expression. These findings support the therapeutic potential of CDK inhibition in neuroblastoma and further highlight differences in the biological responses elicited by selective and broader CDK inhibitors. Exploratory transcriptomic profiling complemented the functional analyses by identifying candidate expression patterns involving cell-cycle regulation, DNA replication, and mitotic progression. Although these transcriptomic observations require validation in biologically replicated studies, they provide a valuable framework for future mechanistic investigations into MYCN-associated responses to CDK inhibition. Collectively, our findings support continued investigation of CDK-targeted therapies and offer a foundation for future studies aimed at understanding the molecular basis of treatment response in neuroblastoma.

## Figures and Tables

**Table 1:** Comparison of *CDK4* and *CDK6* expression changes measured by RNA sequencing and quantitative real-time PCR.

		*CDK4*		*CDK6*	
**Cell line**	Compound	log2 fold change Real-Time PCR	log2 fold change RNA-Seq	log2 fold change Real-Time PCR	log2 fold change RNA-Seq
**SH-SY5Y**	Abemaciclib	−1.34	−1.63	−1.12	−1.04
**SH-SY5Y**	Arcyriaflavin A	−1.03	−0.97	−1.01	−0.36
**BE(2)-C**	Abemaciclib	−1.54	−1.18	−1.95	−2.44
**BE(2)-C**	Arcyriaflavin A	−1.21	−1.02	−1.12	−1.20

**Table 2:** Primer sequences used in qRT-PCR.

Gene	Forward	Reverse
*GAPDH*	GACCACTTTGTCAAGCTCATTTC	CTCTCTTCCTCTTGTGCTCTTGC
*CDK4*	ATGGCTACCTCTCGATATGAGC	CATTGGGGACTCTCACACTCT
*CDK6*	CCAGGCAGGCTTTTCATTCA	AGGTCCTGGAAGTATGGGTG

## Data Availability

The raw data supporting the conclusions of this article will be made available by the authors on request.

## Supplementary Materials

The following supporting information can be downloaded at https://www.mdpi.com/article/10.3390/ijms27167349/s1.

## Abbreviations

The following abbreviations are used in this manuscript:

CDK	Cyclin-dependent kinase
ANXA5	Annexin V
