# Supplementary material for: Comparative Functional Effects of Abemaciclib and Arcyriaflavin A in MYCN-Amplified and Non-Amplified Neuroblastoma Cells: An Exploratory Transcriptomic Study
Source: Int J Mol Sci. 2026 Aug 17;27(16):7349. doi: 10.3390/ijms27167349 (PMC13513924; doi:10.3390/ijms27167349)
Supplement: Supplementary file 1 [file ijms-27-07349-s001.zip › ijms-4468158-supplementary.pdf]

Supplementary Materials

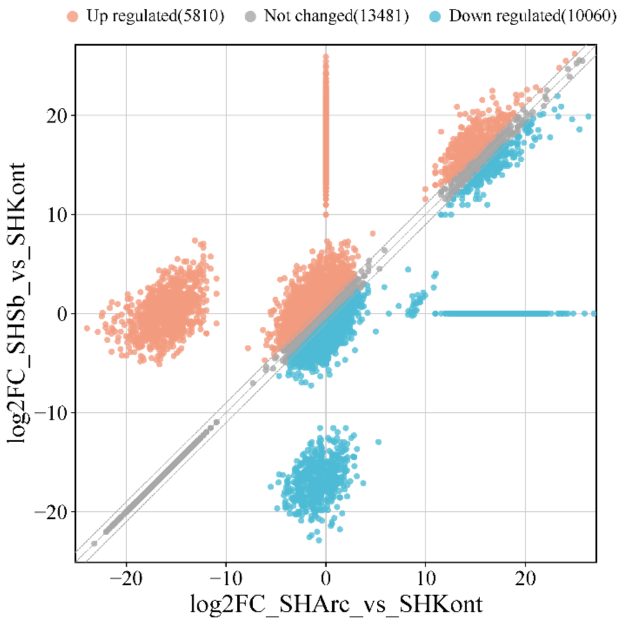

Figure S1. Comparative distribution plot of log_2 fold change (log_2FC) values illustrating gene expression alterations in SH-SY5Y cells treated with Abemaciclib or Arcyriaflavin A relative to the control group. SHSb denotes Abemaciclib-treated SH-SY5Y cells, whereas SHArc denotes Arcyriaflavin A–treated SH-SY5Y cells

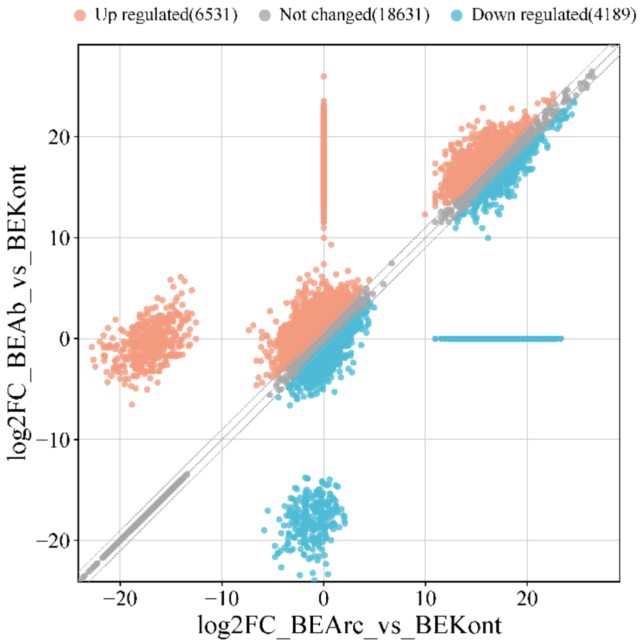

Figure S2. Comparative distribution of log_2 fold change (log_2FC) values illustrating gene expression alterations in BE(2)-C cells treated with Abemaciclib or Arcyriaflavin A relative to the control group.

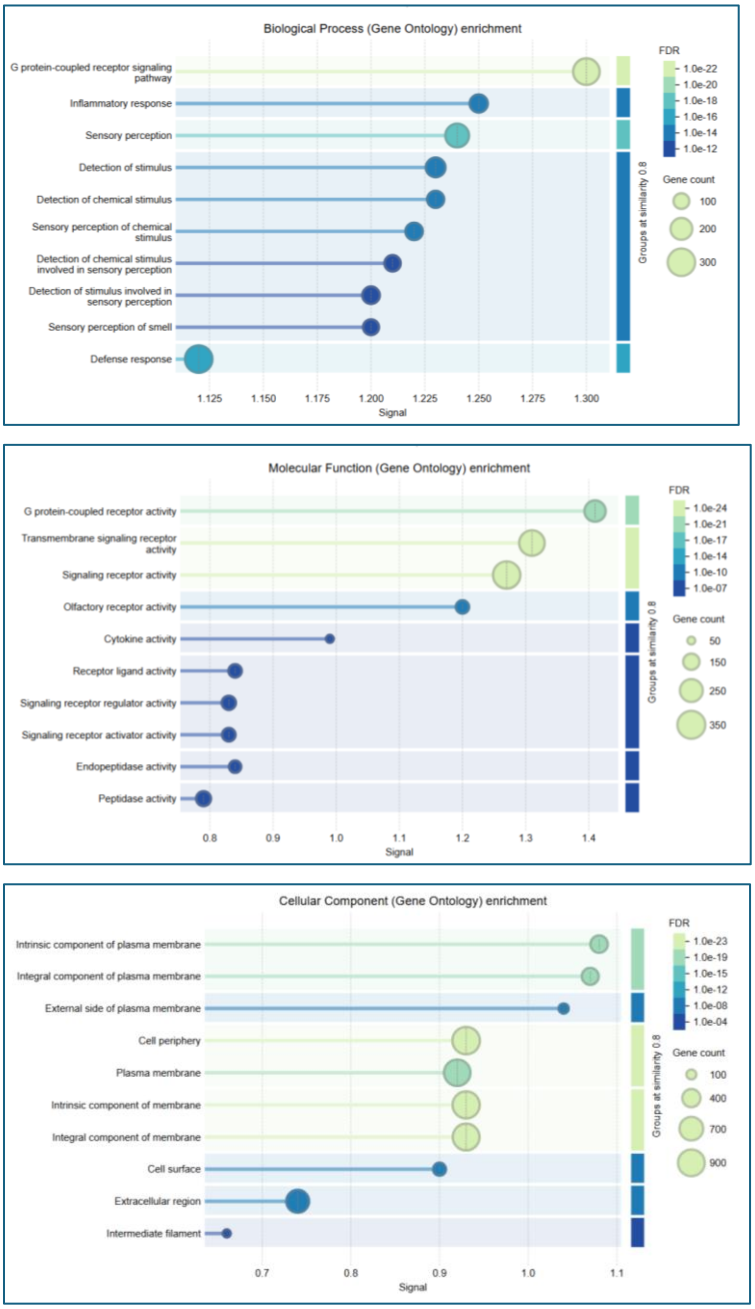

Figure S3. Gene Ontology (GO) enrichment analysis of upregulated genes in SH-SY5Y cells treated with Abemaciclib.

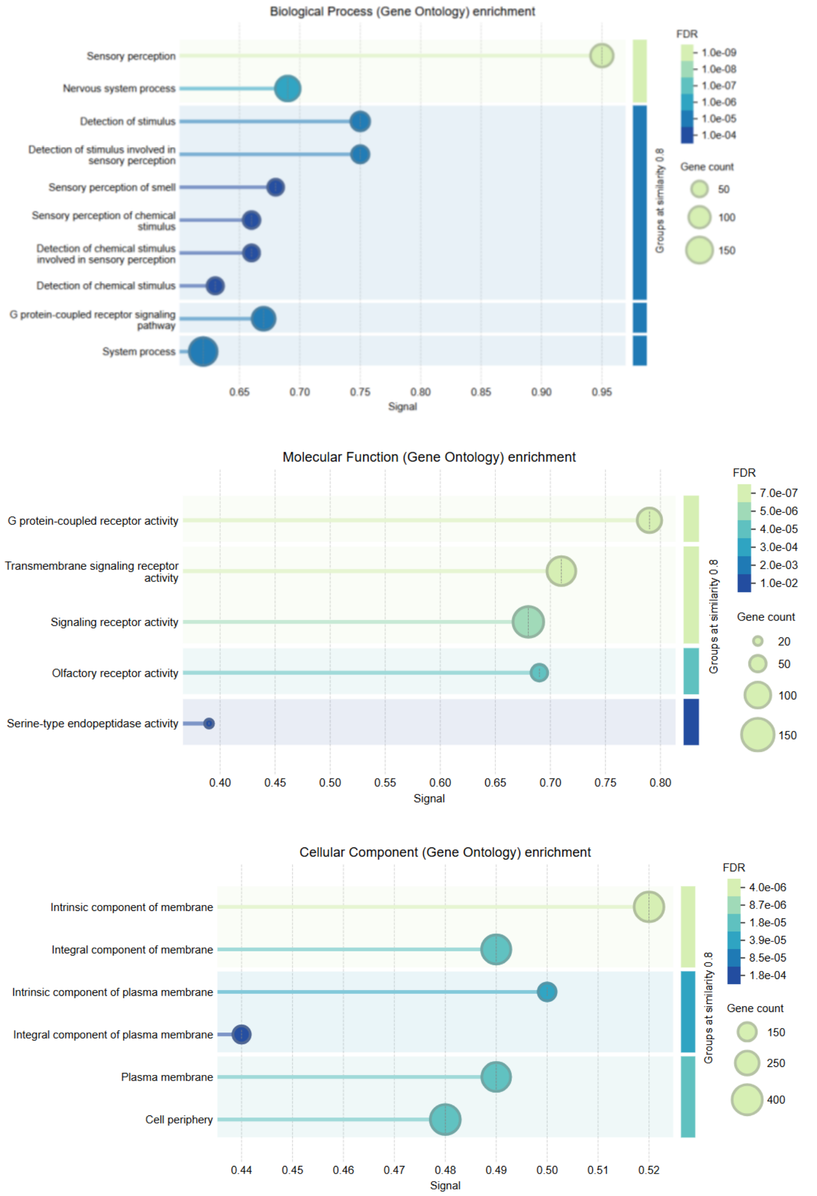

Figure S4. Gene Ontology (GO) enrichment analysis of upregulated genes in SH-SY5Y cells treated with Arcyriaflavin A.

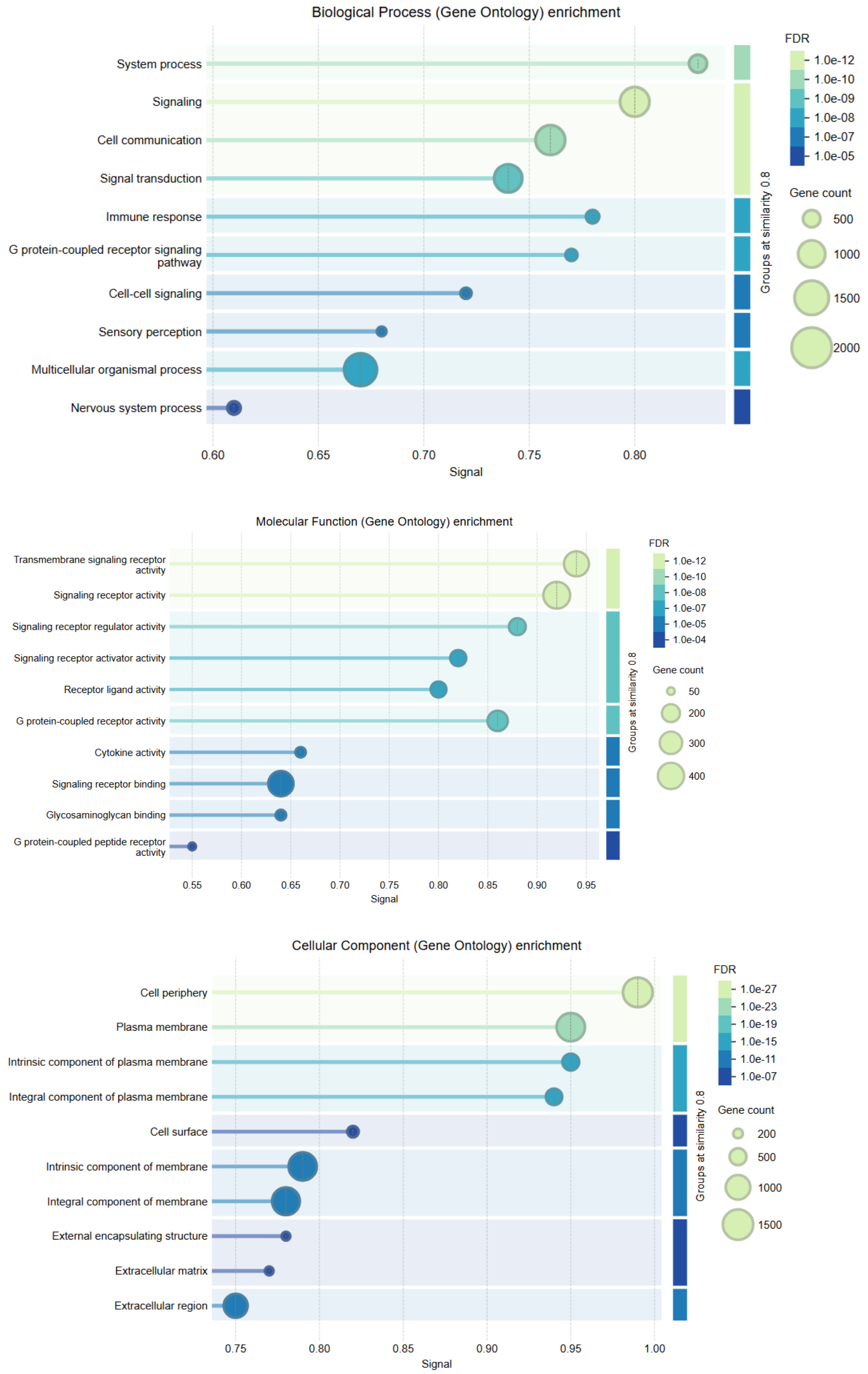

Figure S5. Gene Ontology (GO) enrichment analysis of upregulated genes in BE(2)-C cells treated with Abemaciclib.

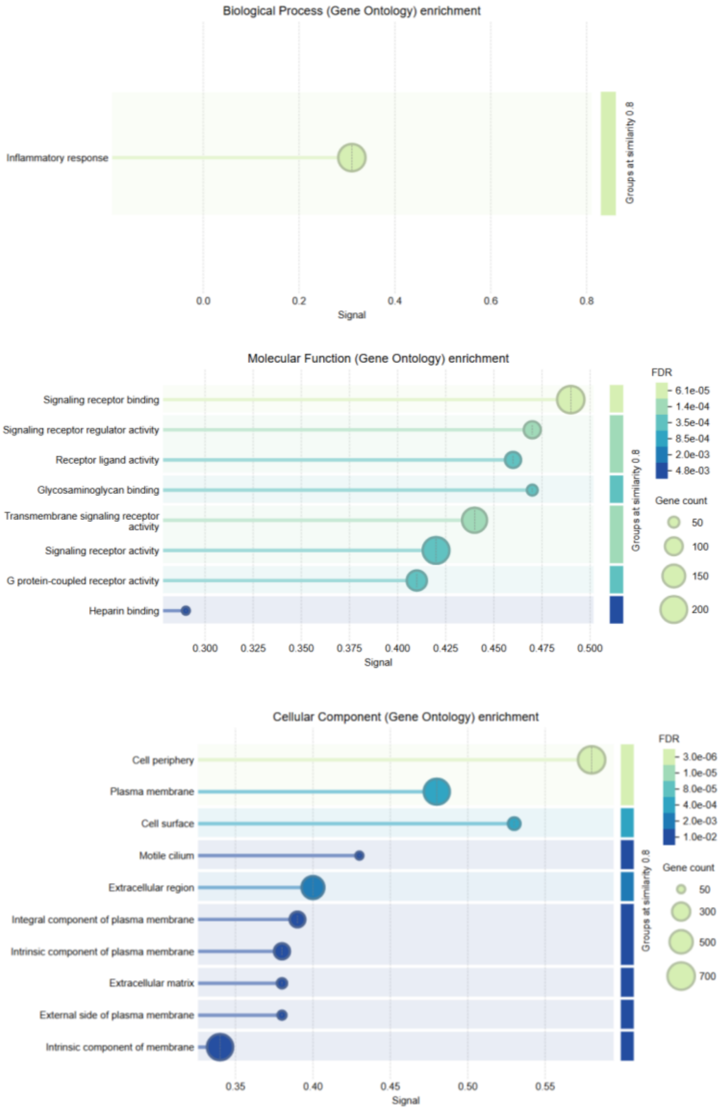

Figure S6. Gene Ontology (GO) enrichment analysis of upregulated genes in BE(2)-C cells treated with Arcyriaflavin A.

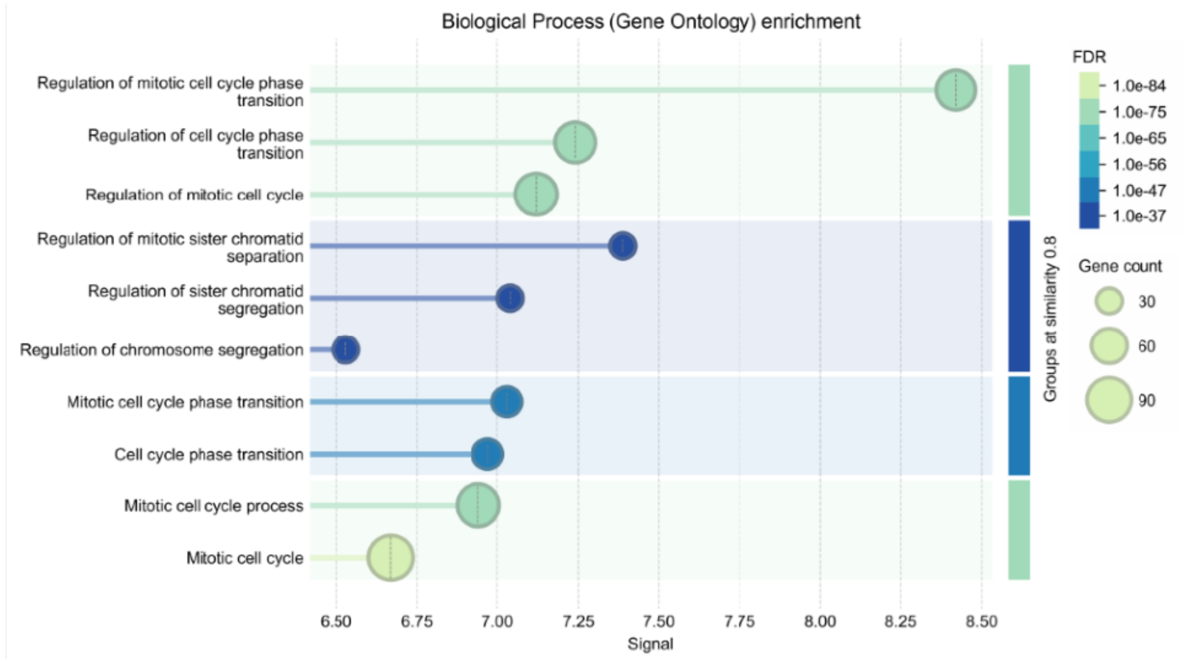

Figure S7. Gene Ontology (GO) Biological Process enrichment analysis. Top enriched GO Biological Process terms among differentially expressed genes. The strongest enrichment was observed for mitotic cell cycle, DNA replication, chromosome segregation, and regulation of cell cycle phase transition, supporting global transcriptional repression of proliferative machinery.

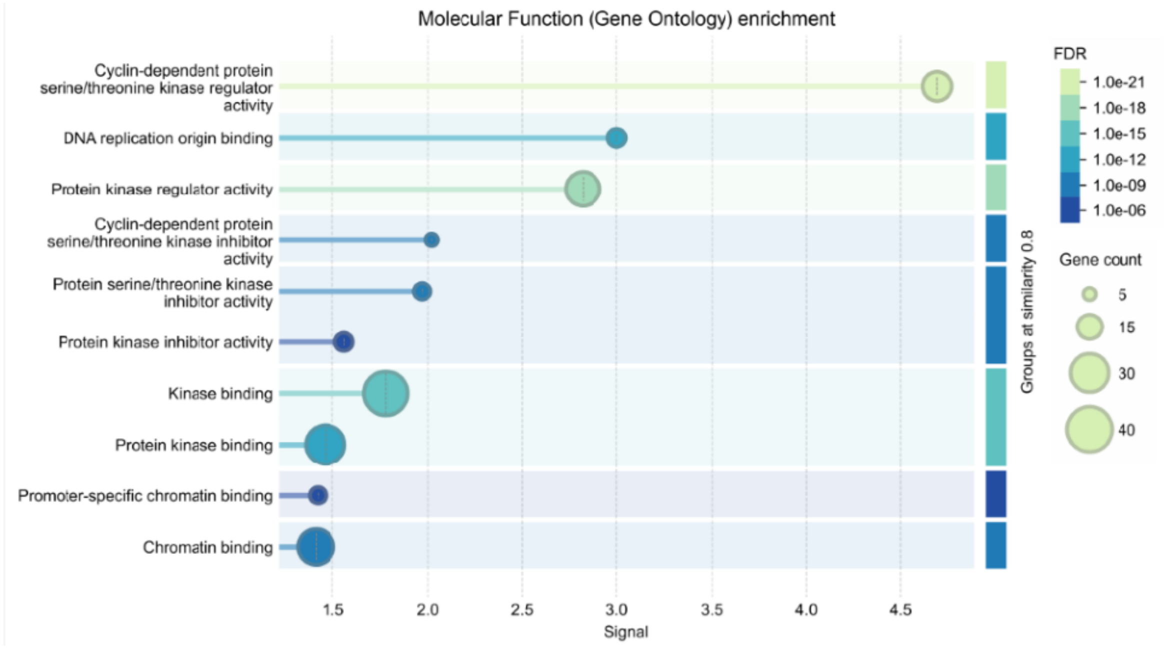

Figure S8. Gene Ontology (GO) Molecular Function enrichment analysis. GO Molecular Function analysis revealed significant enrichment for cyclin-dependent protein kinase activity, DNA-binding transcription factor binding, ATP-dependent helicase activity, and chromatin binding, consistent with inhibition of CDK-driven transcriptional and replication-associated processes.

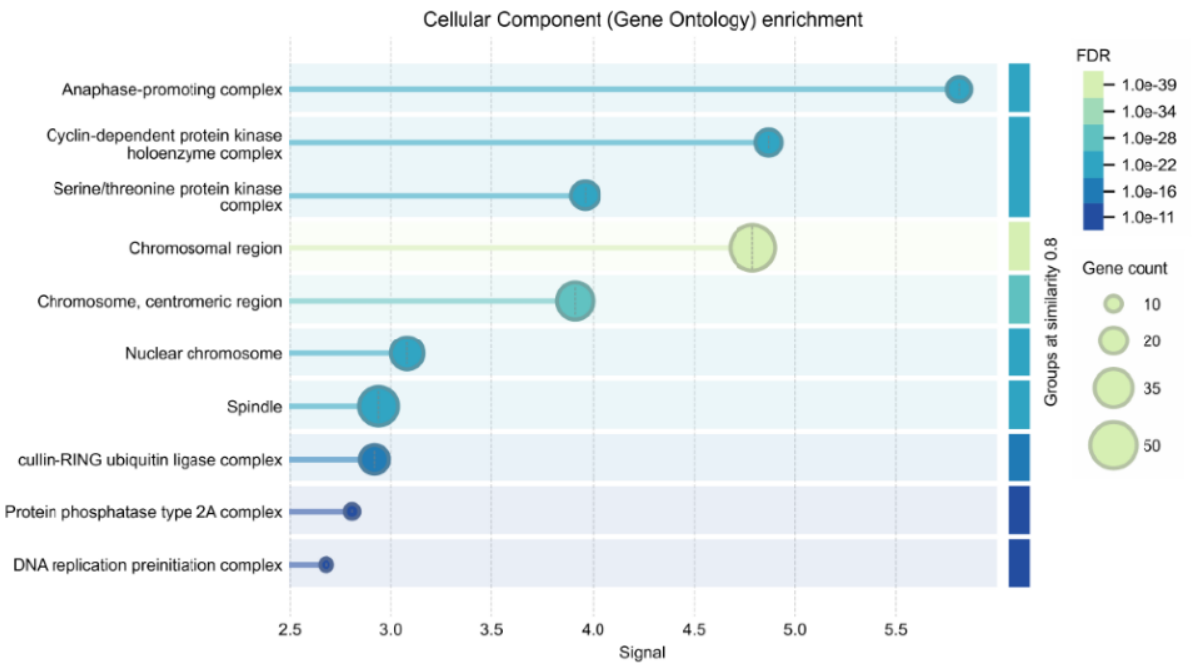

Figure S9. Gene Ontology (GO) Cellular Component enrichment analysis. GO Cellular Component enrichment highlighted overrepresentation of genes localized to the nuclear chromatin, replication fork, spindle apparatus, and anaphase-promoting complex, indicating disruption of mitotic machinery and replication complexes.

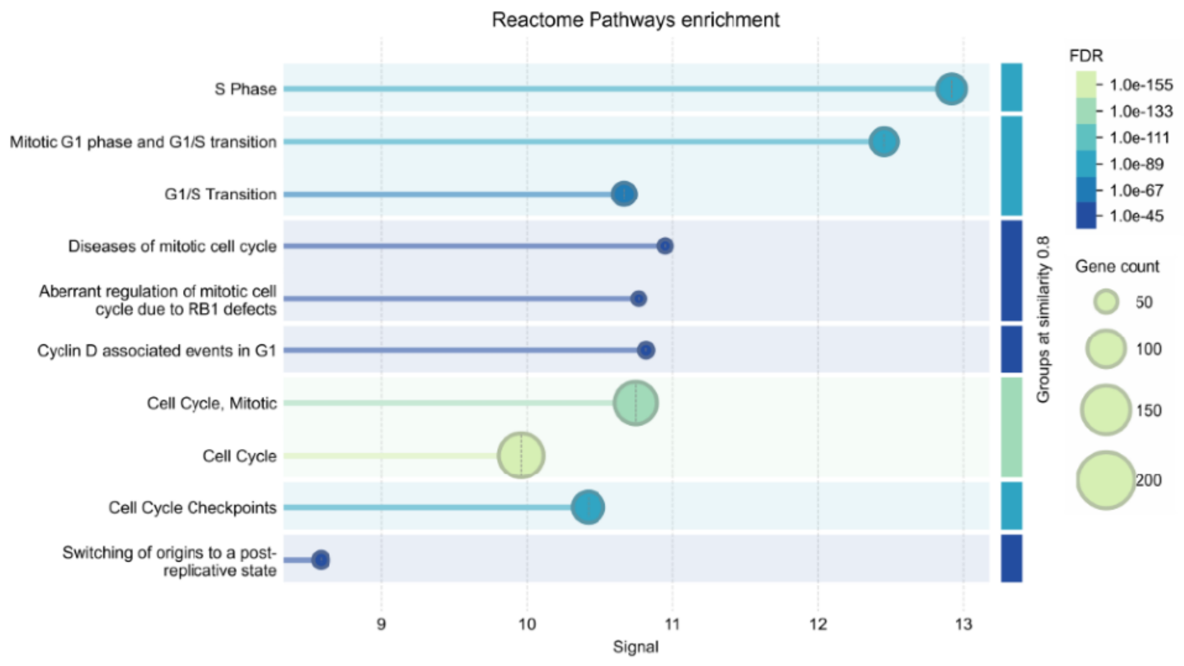

Figure S10. Reactome pathway enrichment analysis. Significant enrichment was observed in pathways related to G1/S transition, E2F-mediated transcription, DNA replication initiation, and mitotic spindle checkpoint control, reinforcing the coordinated suppression of proliferative signaling networks.

Table S1. Fold changes of the genes involved in CDK signaling, G1/S transition, DNA replication licensing, and mitotic progression generally showed reduced expression following treatment with Abemaciclib and Arcyriaflavin A

		BE2C		SH-SY5Y	
**Gene symbol**	**Gene Name**	**Abemaciclib**	**Arcyriaflavin A**	**Abemaciclib**	**Arcyriaflavin A**
**CDK2**	cyclin dependent kinase 2	-2.295455884	-0.494155648	-1.201197774	-0.869473426
**CDK4**	cyclin dependent kinase 4	-1.60090264	-0.95183758	-1.158844252	-1.003362301
**CDK6**	cyclin dependent kinase 6	-1.023582935	-0.352234468	-2.239586647	-1.140993772
**CDK7**	cyclin dependent kinase 7	0.599654674	-0.265424825	-0.704396362	0.12683458
**CDKN1A**	cyclin dependent kinase inhibitor 1A [KO:K06625]	-2.414706112	-1.025832518	-0.220424575	-1.112949905
**CDKN1B**	cyclin dependent kinase inhibitor 1B [KO:K06624]	-0.264381775	-0.236064686	-1.602533764	-0.302779547
**STAG1**	STAG1 cohesin complex component [KO:K06671]	-0.029031075	0.173096491	-1.422515976	-0.641842611
**CDKN1C**	cyclin dependent kinase inhibitor 1C [KO:K09993]	-0.104579763	-0.054494531	2.735622928	-0.887105944
**CDKN2A**	cyclin dependent kinase inhibitor 2A [KO:K06621]	-1.13706626	-0.301598189	0.010338448	-0.508271898
**CDKN2B**	cyclin dependent kinase inhibitor 2B [KO:K04685]	0.181262251	0.403597816	-1.174818698	-0.282428842
**CDKN2C**	cyclin dependent kinase inhibitor 2C [KO:K06622]	-0.623546558	-0.288242036	-2.289394206	-0.977695126
**CDKN2D**	cyclin dependent kinase inhibitor 2D [KO:K06623]	-0.599977841	-0.200499815	-0.646839764	0.199344171
**ANAPC10**	anaphase promoting complex subunit 10 [KO:K03357]	-0.59731697	0.049206508	-1.219247706	-0.429312939
**NDC80**	NDC80 kinetochore complex component [KO:K11547]	-1.866274949	-0.084384571	-1.508860505	-0.188349596
**MAD2L2**	mitotic arrest deficient 2 like 2 [KO:K13728]	-0.944290567	-0.122730533	-1.348313812	-1.070974642
**STAG2**	STAG2 cohesin complex component [KO:K06671]	-0.442544607	0.241455183	-1.991967966	-1.208930463
**PTTG2**	pituitary tumor-transforming 2 [KO:K06635]	-2.244140094	-1.086062599	-1.710767621	-0.748425747

**GADD45G**	growth arrest and DNA damage inducible gamma [KO:K04402]	-0.108934372	-0.618778886	3.599420093	0.036977858
**DBF4**	DBF4-CDC7 kinase regulatory subunit [KO:K06629]	0.022013742	0.671787	-1.86568009	-0.004240442
**YWHAQ**	tyrosine 3-monooxygenase/ tryptophan 5-monooxygenase activation protein theta [KO:K16197]	-0.839461401	0.164845154	-1.349170801	-0.331379198
**CHEK1**	checkpoint kinase 1	-1.064596048	-0.274576485	-1.569780755	-0.345387068
**CHEK2**	checkpoint kinase 2	-2.09016157	-1.236012437	-2.295523964	-1.025307775
**CDCA5**	cell division cycle associated 5 [KO:K17390]	-0.818913559	-1.006481876	-0.26276447	-0.568794434
**ESCO1**	establishment of sister chromatid cohesion N-acetyltransferase 1 [KO:K11268] [EC:2.3.1.-]	0.531762307	0.297994813	-1.588528114	-0.649050531
**ANAPC16**	anaphase promoting complex subunit 16 [KO:K25229]	-1.00513339	-0.396931966	-1.25943638	0.015629135
**CREBBP**	CREB binding protein	0.488108035	0.948534096	-2.243242378	-1.146545158
**SGO1**	shugoshin 1 [KO:K11580]	-1.492863226	-0.354514401	-1.632142203	-0.233637726
**ESCO2**	establishment of sister chromatid cohesion N-acetyltransferase 2 [KO:K11268] [EC:2.3.1.-]	-2.635118713	-0.122712734	-1.774111893	-0.663263583
**GADD45A**	growth arrest and DNA damage inducible alpha [KO:K04402]	0.290930665	0.125283776	0.685781296	0.495752949
**DDX11**	DEAD/H-box helicase 11	-2.293056889	-0.958777337	-1.30283812	-0.689067108
**CDC14C**	cell division cycle 14C	-0.388959672	-0.076605508	-0.659371059	-0.763598428
**E2F1**	E2F transcription factor 1 [KO:K17454]	-3.00829068	-0.641913672	-1.276861677	-0.519146219
**E2F2**	E2F transcription factor 2 [KO:K09389]	-2.605022724	-0.805227793	-1.665061614	-1.219382806
**E2F3**	E2F transcription factor 3 [KO:K06620]	-0.457238175	0.074546953	-0.579418195	-0.488748413
**E2F4**	E2F transcription factor 4 [KO:K04682]	-0.304332565	-0.530773418	-1.211508844	-0.599964193

**E2F5**	E2F transcription factor 5 [KO:K04682]	-1.205104223	-0.830660301	-1.616120149	-0.601977841
**EP300**	E1A binding protein p300	0.259725808	-0.147811367	-1.983426043	-0.491578981
**PDS5B**	PDS5 cohesin associated factor B [KO:K11267]	0.07098188	0.213284468	-1.689665086	-0.184634918
**WAPL**	WAPL cohesin release factor [KO:K25163]	-0.255998516	-0.107300287	-1.626272161	-0.294314121
**PDS5A**	PDS5 cohesin associated factor A [KO:K11267]	-0.521017863	0.111559865	-1.556610744	-0.73543415
**MAU2**	MAU2 sister chromatid cohesion factor [KO:K11266]	0.101102305	-0.147749713	-1.739928656	-0.608419883
**ORC6**	origin recognition complex subunit 6 [KO:K02608]	-0.982259584	-0.533733108	-0.936582239	-0.127569401
**ORC3**	origin recognition complex subunit 3 [KO:K02605]	0.185336556	0.109449655	-1.12716981	-0.326991431
**CDC26**	cell division cycle 26 [KO:K03359]	0.147957881	0.00012	-0.045453818	-0.068018423
**ABL1**	ABL proto-oncogene 1, non-receptor tyrosine kinase	-0.414001381	0.125940405	-1.896206104	-0.685755519
**NIPBL**	NIPBL cohesin loading factor [KO:K06672]	-0.193588466	-0.004025793	-1.570342711	-0.07869533
**ANAPC13**	anaphase promoting complex subunit 13 [KO:K12456]	0.073476514	0.432868395	-1.930967752	-0.519059818
**ANAPC15**	anaphase promoting complex subunit 15 [KO:K25228]	-1.498446562	-0.797277962	-0.211203198	-1.459881704
**FBXO5**	F-box protein 5 [KO:K10292]	-1.68443386	-0.345888566	-0.91956983	-0.369750371
**MTBP**	MDM2 binding protein [KO:K26116]	-0.394473227	-0.137667662	-1.598634641	-0.33589675
**SMC1B**	structural maintenance of chromosomes 1B [KO:K06636]	-0.038438798	-0.05057936	-0.008933125	-0.055769539
**SFN**	stratifin [KO:K06644]	0.048236186	0.0002	-0.172836597	-0.167599948
**GSK3B**	glycogen synthase kinase 3 beta [KO:K03083] [EC:2.7.11.26]	-0.478438248	-0.413385374	-1.605191915	-0.367078051
**ANAPC2**	anaphase promoting complex subunit 2 [KO:K03349]	-0.496879812	-0.463441227	-1.534139744	-0.509903513
**ANAPC4**	anaphase promoting	-0.17902738	-0.150607348	-1.943873476	-0.518579219

	complex subunit 4 [KO:K03351]				
**HDAC1**	histone deacetylase 1 [KO:K06067] [EC:3.5.1.98]	0.217156386	-0.489242188	-0.659981431	-1.106125074
**HDAC2**	histone deacetylase 2 [KO:K06067] [EC:3.5.1.98]	-0.816535945	-0.063088429	-1.807330656	-0.56264128
**MAD2L1**	mitotic arrest deficient 2 like 1 [KO:K02537]	-2.558270797	-0.873489729	-1.653766023	-0.23065115
**SMAD2**	SMAD family member 2 [KO:K04500]	0.235458293	0.407511357	-1.113939372	-0.449509507
**SMAD3**	SMAD family member 3 [KO:K23605]	-0.475165995	-0.127487819	-1.225091508	-0.681129
**SMAD4**	SMAD family member 4 [KO:K04501]	-0.227723211	-0.231699584	-1.063123174	-0.321440874
**MCM2**	minichromosome maintenance complex component 2 [KO:K02540] [EC:5.6.2.3]	-2.004458238	-0.45417712	-1.609299707	-0.974928199
**MCM3**	minichromosome maintenance complex component 3 [KO:K02541] [EC:5.6.2.3]	-1.009923482	-0.23358609	-1.715082476	-0.419214359
**MCM4**	minichromosome maintenance complex component 4 [KO:K02212] [EC:5.6.2.3]	-2.526709296	-0.50336323	-1.791394667	-0.683895083
**MCM5**	minichromosome maintenance complex component 5 [KO:K02209] [EC:5.6.2.3]	-2.03271585	-0.95935635	-1.712351048	-0.418220774
**MCM6**	minichromosome maintenance complex component 6 [KO:K02542] [EC:5.6.2.3]	-2.554553717	-0.51492122	-1.861221794	-0.603814429
**MCM7**	minichromosome maintenance complex component 7 [KO:K02210] [EC:5.6.2.3]	-2.22811372	-0.672443409	-1.490038062	-0.61088602
**MDM2**	MDM2 proto-oncogene [KO:K06643] [EC:2.3.2.27]	-0.502154886	0.032978399	-2.267277052	-0.703909945
**MYC**	MYC proto-oncogene, bHLH transcription	2.165574577	1.040893589	-2.683834755	-0.073989469

	factor [KO:K04377]				
**GADD45B**	growth arrest and DNA damage inducible beta [KO:K04402]	-0.153584317	-0.731083298	4.503290736	-0.169498655
**ATM**	ATM serine/threonine kinase [KO:K04728] [EC:2.7.11.1]	-0.215465839	0.059701802	-1.842163882	-0.687251782
**WEE2**	WEE2 oocyte meiosis inhibiting kinase [KO:K06632] [EC:2.7.11.1]	0.085424656	0.10433666	-0.044781715	0.027298734
**ORC1**	origin recognition complex subunit 1 [KO:K02603]	-1.364746911	-0.634824061	-1.409909665	-0.54002964
**ORC2**	origin recognition complex subunit 2 [KO:K02604]	-0.071361095	0.295916617	-2.042808846	-0.69098098
**ORC4**	origin recognition complex subunit 4 [KO:K02606]	-0.355198091	-0.081994037	-2.182138589	-0.838902004
**ORC5**	origin recognition complex subunit 5 [KO:K02607]	-0.560416457	-0.369409782	-1.308310762	-0.018893247
**PCNA**	proliferating cell nuclear antigen [KO:K04802]	-2.5750143	0.068457936	-2.112696267	-0.265015237
**FZR1**	fizzy and cell division cycle 20 related 1 [KO:K03364]	0.144164096	-0.090307629	-1.720489149	-0.486981989
**ANAPC5**	anaphase promoting complex subunit 5 [KO:K03352]	-1.468173081	-0.345957058	-1.582231393	-0.776191744
**ANAPC7**	anaphase promoting complex subunit 7 [KO:K03354]	-1.217957486	-0.234588014	-2.03438276	-0.310694787
**ANAPC11**	anaphase promoting complex subunit 11 [KO:K03358]	-0.515571623	0.009853167	-0.640255748	-0.620888122
**PLK1**	polo like kinase 1 [KO:K06631] [EC:2.7.11.21]	-2.166898242	-0.553270041	-2.057479666	-0.419163276
**ATR**	ATR serine/threonine kinase [KO:K06640] [EC:2.7.11.1]	-0.330153328	0.105394159	-1.131493919	-0.178426818
**ATRX**	ATRX chromatin remodeler [KO:K10779]	0.290289049	0.558319518	-1.964006118	-0.553332533
**PPP2CA**	protein phosphatase 2	-0.772346125	-0.452557696	-0.948252797	-0.322998234

	catalytic subunit alpha [KO:K04382] [EC:3.1.3.16]				
**PPP2CB**	protein phosphatase 2 catalytic subunit beta	-0.227681443	-0.2001144	-1.006946211	-0.801454321
**PPP2R1A**	protein phosphatase 2 scaffold subunit Aalpha [KO:K03456]	-0.684735882	-0.496268961	-0.229737477	-0.850104126
**PPP2R1B**	protein phosphatase 2 scaffold subunit Abeta [KO:K03456]	-0.747612838	0.002815016	-1.823823357	-0.680334634
**PPP2R5A**	protein phosphatase 2 regulatory subunit B'alpha [KO:K11584]	0.023096971	-0.111345525	-1.521435837	-0.26700604
**PPP2R5B**	protein phosphatase 2 regulatory subunit B'beta [KO:K11584]	0.208671582	0.029419436	-0.749331252	-0.910365944
**PPP2R5C**	protein phosphatase 2 regulatory subunit B'gamma [KO:K11584]	-0.677459843	-0.385758245	-1.307141137	-0.689967904
**PPP2R5D**	protein phosphatase 2 regulatory subunit B'delta [KO:K11584]	-0.294651058	0.161108165	-1.50252821	-0.097236279
**PPP2R5E**	protein phosphatase 2 regulatory subunit B'epsilon [KO:K11584]	-0.094900277	0.063638641	-1.475512632	-0.282334398
**HDAC8**	histone deacetylase 8 [KO:K11405] [EC:3.5.1.98]	-0.878460455	-0.34705487	-1.280862218	-0.378967435
**PRKDC**	protein kinase, DNA-activated, catalytic subunit [KO:K06642] [EC:2.7.11.1]	-1.278648468	-0.232872415	-1.534731616	-0.718785673
**KNL1**	kinetochore scaffold 1 [KO:K11542]	-1.749994113	0.493691253	-2.249266025	-0.2213679
**RAD21**	RAD21 cohesin complex component [KO:K06670]	-0.697582638	-0.097682428	-1.596310046	-0.314132145
**RB1**	RB transcriptional corepressor 1 [KO:K06618]	-0.745210506	-0.159138804	-0.865021852	-0.35872503
**RBL1**	RB transcriptional	-1.869365142	-1.053084535	-1.524190917	0.163642456

	corepressor like 1 [KO:K04681]				
**RBL2**	RB transcriptional corepressor like 2 [KO:K16332]	-0.228332502	-0.617371348	-1.365285822	-0.251397581
**CCND1**	cyclin D1 [KO:K04503]	-0.127863765	0.34059043	-1.068133799	-2.39144107
**ANAPC1**	anaphase promoting complex subunit 1 [KO:K03348]	-0.699385934	-0.340598056	-2.190659139	-0.554571298
**SKP1**	S-phase kinase associated protein 1 [KO:K03094]	-0.804217157	-0.401190813	-0.268764195	-0.043940135
**SKP2**	S-phase kinase associated protein 2 [KO:K03875]	-0.922225516	-0.337538998	-0.615487277	-0.663874191
**BUB1**	BUB1 mitotic checkpoint serine/threonine kinase [KO:K02178] [EC:2.7.11.1]	-1.497659402	-0.034150545	-2.207706258	-0.413810173
**BUB1B**	BUB1 mitotic checkpoint serine/threonine kinase B [KO:K06637] [EC:2.7.11.1]	-1.647058457	-0.208237653	-1.935805821	-0.815060384
**TFDP1**	transcription factor Dp-1 [KO:K04683]	-1.479918464	-0.345565082	-0.665319048	-1.15343907
**TFDP2**	transcription factor Dp-2 [KO:K09392]	-0.615789171	0.581797353	-2.300175754	-0.268677551
**TGFB1**	transforming growth factor beta 1 [KO:K13375]	-0.722652701	0.087340116	-1.271819659	-1.589699067
**TGFB2**	transforming growth factor beta 2 [KO:K13376]	0.663968161	-0.10773024	0.203150625	0.291356396
**TGFB3**	transforming growth factor beta 3 [KO:K13377]	-0.131269959	-0.392083845	-1.11747483	-0.734006483
**TP53**	tumor protein p53 [KO:K04451]	-1.650861348	-0.89667979	-1.100506259	-0.742952281
**TTK;**	TTK protein kinase [KO:K08866] [EC:2.7.12.1]	-0.563494292	0.736936366	-2.055661332	-0.225537116
**WEE1**	WEE1 G2 checkpoint kinase [KO:K06632] [EC:2.7.11.1]	-1.561548576	-0.477233452	-0.783219427	0.113719373
**YWHAB**	tyrosine 3-monooxygenase/ tryptophan 5-monooxygenase	-0.640467599	0.25299983	-1.356679774	-0.477694355

	activation protein beta [KO:K16197]				
**YWHAE**	tyrosine 3-monooxygenase/ tryptophan 5-monooxygenase activation protein epsilon [KO:K06630]	-0.497456134	-0.007716988	-0.955772911	-0.681541256
**YWHAG**	tyrosine 3-monooxygenase/ tryptophan 5-monooxygenase activation protein gamma [KO:K16198]	-0.609464732	-0.154924021	-1.388431849	-0.487314515
**YWHAH**	tyrosine 3-monooxygenase/ tryptophan 5-monooxygenase activation protein eta [KO:K16198]	-1.214298564	0.016507345	-0.986484618	0.112834988
**YWHAZ**	tyrosine 3-monooxygenase/ tryptophan 5-monooxygenase activation protein zeta [KO:K16197]	-0.818440643	-0.298874168	-1.450842491	-0.460055176
**ZBTB17**	zinc finger and BTB domain containing 17 [KO:K10500]	0.174951995	0.032779682	-0.464047021	-0.55099645
**DBF4B**	DBF4B-CDC7 kinase regulatory subunit [KO:K06629]	-1.026715875	-0.863650285	-1.51931876	-0.722957155
**CDT1**	chromatin licensing and DNA replication factor 1 [KO:K10727]	-1.210108283	-0.850662503	-1.676841037	-0.810830586
**SMC1A**	structural maintenance of chromosomes 1A [KO:K06636]	-0.379843768	0.706036736	-1.060227411	-0.132095206
**CDC7**	cell division cycle 7 [KO:K02214] [EC:2.7.11.1]	-1.082392715	-0.432723306	-1.608247019	-0.394095743
**CDC45**	cell division cycle 45 [KO:K06628]	-2.996662183	-0.661940088	-1.641238958	-0.555494259
**MAD1L1**	mitotic arrest deficient 1 like 1 [KO:K06679]	-0.276060494	0.396203665	-1.154712381	-0.238806108
**CUL1**	cullin 1 [KO:K03347]	0.180332631	0.960609662	-1.908177839	-0.290711021
**CCNB3**	cyclin B3 [KO:K21771]	-0.060330534	-0.103321468	-0.525750722	-0.204738915
**CDC14B**	cell division cycle 14B [KO:K06639]	-0.315087423	-0.125231702	-1.671155151	-1.082625652

	[EC:3.1.3.16 3.1.3.48]				
**CDC14A**	cell division cycle 14A [KO:K06639] [EC:3.1.3.16 3.1.3.48]	0.473507296	-0.292214895	-1.044642402	-0.693406275
**CDC23**	cell division cycle 23 [KO:K03355]	-0.593364013	-0.299795481	-0.979512608	-0.435674158
**CDC16**	cell division cycle 16 [KO:K03353]	-0.177343411	0.095297572	-1.663973714	-0.355286968
**CCNA2**	cyclin A2 [KO:K06627]	-2.587113954	-0.791599785	-2.471069929	-0.527157363
**CCNA1**	cyclin A1 [KO:K06627]	-0.112366523	-0.095157233	0.020989515	-0.127341039
**CCNB1**	cyclin B1 [KO:K05868]	-1.59509049	-0.459089838	-2.108088542	-0.886214953
**CCND2**	cyclin D2 [KO:K10151]	0.014355293	0.01720929	-0.794533281	0.118623091
**CCND3**	cyclin D3 [KO:K10152]	-0.53688123	-0.117899244	-1.059607056	-0.682840323
**CCNE1**	cyclin E1 [KO:K06626]	-1.631011927	0.041667824	-1.4695603	-0.598877075
**CCNH**	cyclin H [KO:K06634]	-0.247634827	-0.222138598	-1.797683187	-0.53174459
**TICRR**	TOPBP1 interacting checkpoint and replication regulator [KO:K26117]	-1.414359382	-0.47528838	-1.168305354	-0.493655148
**PKMYT1**	protein kinase, membrane associated tyrosine/threonine 1 [KO:K06633] [EC:2.7.11.1]	-3.134504807	-1.15895411	-2.204827277	-1.436892373
**SMC3**	structural maintenance of chromosomes 3 [KO:K06669]	-0.503735829	0.426715649	-1.941705902	-0.256976019
**CCNB2**	cyclin B2 [KO:K21770]	-1.343462961	-0.26369415	-1.986716268	-0.297502206
**CCNE2**	cyclin E2 [KO:K06626]	-3.097189708	-0.060172899	-1.597950938	-0.820502911
**BUB3**	BUB3 mitotic checkpoint protein [KO:K02180]	-1.556088163	-0.646680094	-1.941809638	-0.605834377
**AURKB**	aurora kinase B [KO:K11479] [EC:2.7.11.1]	-3.165030909	-1.140459326	-1.844124374	-0.797405919
**PTTG1**	PTTG1 regulator of sister chromatid separation, securin [KO:K06635]	-0.758406518	0.679297062	-2.424098456	-0.437155153
**TRIP13**	thyroid hormone receptor interactor 13 [KO:K22399]	-1.844641466	-0.171209323	-2.087965571	-1.458549472

**MAD2L1BP**	MAD2L1 binding protein [KO:K26093]	-0.515910528	-0.204174314	-0.578285142	-0.636133442
**ESPL1**	extra spindle pole bodies like 1, separase [KO:K02365] [EC:3.4.22.49]	-2.937399314	-0.884890429	-2.095910032	-0.714257277
**CDK1**	cyclin dependent kinase 1 [KO:K02087] [EC:2.7.11.22 2.7.11.23]	-1.817424112	-0.522724208	-2.00738704	-0.735045418
**CDC6**	cell division cycle 6 [KO:K02213]	-1.779995993	0.024397129	-1.923331121	-0.835909805
**CDC20**	cell division cycle 20 [KO:K03363]	-1.959300445	-0.800161999	-2.410848241	-0.797578414
**CDC25A**	cell division cycle 25A	-2.712578632	-0.603442384	-1.013326904	-0.786673069
**CDC25B**	cell division cycle 25B	-1.973735066	-0.860668721	-1.433999644	-0.943259979
**CDC25C**	cell division cycle 25C	-1.156664914	-0.588045842	-1.607577824	-0.385951774
**CDC27**	cell division cycle 27	-0.61541183	-0.088039402	-1.626857697	-1.189025552
**RBX1**	ring-box 1	-0.966077312	-0.417666074	-1.775603654	-0.658987635
**MYCN**	MYCN proto-oncogene, bHLH transcription factor	-2.44006743	-0.535401519	-0.279205697	-0.212471708
**MYC**	MYC proto-oncogene, bHLH transcription factor	2.165574577	1.040893589	-2.683834755	-0.073989469
**E2F4**	E2F transcription factor 4	-0.304332565	-0.530773418	-1.211508844	-0.599964193
**E2F1**	E2F transcription factor 1	-3.00829068	-0.641913672	-1.276861677	-0.519146219
**E2F2**	E2F transcription factor 2	-2.605022724	-0.805227793	-1.665061614	-1.219382806
**E2F6**	E2F transcription factor 6	-0.205388566	-0.452823858	-1.177691884	-0.396225977
**E2F3**	E2F transcription factor 3	-0.457238175	0.074546953	-0.579418195	-0.488748413
**E2F5**	E2F transcription factor 5	-1.205104223	-0.830660301	-1.616120149	-0.601977841
**E2F8**	E2F transcription factor 8	-1.599592773	-0.731673928	-1.938631705	-0.806871267
**E2F7**	E2F transcription factor 7	-2.359780106	0.425425987	-1.275763871	0.097011383
**RB1**	RB transcriptional corepressor 1	-0.745210506	-0.159138804	-0.865021852	-0.35872503
